# Biomphalysin, a New β Pore-forming Toxin Involved in *Biomphalaria glabrata* Immune Defense against *Schistosoma mansoni*

**DOI:** 10.1371/journal.ppat.1003216

**Published:** 2013-03-21

**Authors:** Richard Galinier, Julien Portela, Yves Moné, Jean François Allienne, Hélène Henri, Stéphane Delbecq, Guillaume Mitta, Benjamin Gourbal, David Duval

**Affiliations:** 1 CNRS, UMR 5244, Ecologie et Evolution des Interactions (2EI), Perpignan, France; 2 Université de Perpignan Via Domitia, Perpignan, France; 3 Université de Lyon, Lyon; Université Lyon 1; CNRS, UMR 5558, Laboratoire de Biométrie et Biologie Evolutive, Villeurbanne, France; 4 EA 4558, Vaccination Antiparasitaire, Laboratoire de Biologie Cellulaire et Moléculaire UFR Pharmacie, Montpellier, France; Oregon State University, United States of America

## Abstract

Aerolysins are virulence factors belonging to the β pore-forming toxin (β-PFT) superfamily that are abundantly distributed in bacteria. More rarely, β-PFTs have been described in eukaryotic organisms. Recently, we identified a putative cytolytic protein in the snail, *Biomphalaria glabrata*, whose primary structural features suggest that it could belong to this β-PFT superfamily. In the present paper, we report the molecular cloning and functional characterization of this protein, which we call Biomphalysin, and demonstrate that it is indeed a new eukaryotic β-PFT. We show that, despite weak sequence similarities with aerolysins, Biomphalysin shares a common architecture with proteins belonging to this superfamily. A phylogenetic approach revealed that the gene encoding Biomphalysin could have resulted from horizontal transfer. Its expression is restricted to immune-competent cells and is not induced by parasite challenge. Recombinant Biomphalysin showed hemolytic activity that was greatly enhanced by the plasma compartment of *B. glabrata*. We further demonstrated that Biomphalysin with plasma is highly toxic toward *Schistosoma mansoni* sporocysts. Using *in vitro* binding assays in conjunction with Western blot and immunocytochemistry analyses, we also showed that Biomphalysin binds to parasite membranes. Finally, we showed that, in contrast to what has been reported for most other members of the family, lytic activity of Biomphalysin is not dependent on proteolytic processing. These results provide the first functional description of a mollusk immune effector protein involved in killing *S. mansoni*.

## Author Summary

Schistosomiasis is the second most widespread tropical parasitic disease after malaria. It is caused by flatworms of the genus *Schistosoma*. Its life cycle is complex and requires certain freshwater snail species as intermediate host. Given the limited options for treating *S. mansoni* infections, much research has focused on a better understanding of the immunobiological interactions between the invertebrate host *Biomphalaria glabrata* and its parasite *S. mansoni*. A number of studies published over the last two decades have contributed greatly to our understanding of *B. glabrata* innate immune mechanisms involved in the defense against parasite. However, most studies have focused on the identification of recognition molecules or immune receptors involved in the host/parasite interplay. In the present study, we report the first functional description of a mollusk immune effector protein involved in killing *S. mansoni*, a protein related to the β pore forming toxin that we named Biomphalysin.

## Introduction

Schistosomiasis, or bilharzia, is a tropical disease caused by worms of the genus *Schistosoma*. The main disease-causing species are *Schistosoma haematobium*, *Schistosoma japonicum*, and *Schistosoma mansoni*. An estimated 200 million people in 74 countries suffer from schistosomiasis [1], [2]. The World Health Organization expert committee (WHO Technical Report Series 912: prevention and control of schistosomiasis and soil transmitted helminthiasis (WHO, Geneva, 2002)) concluded that yearly deaths could be as high as 200,000 making schistosomiasis the most severe tropical disease after malaria in terms of mortality [1]. No vaccines are yet available to fight *S. mansoni*, and current chemotherapy relies on a single drug, praziquantel, for which resistant cases have been reported [1], [2].

The life cycle of the parasite requires contamination of surface water by excrement, specific freshwater snails as intermediate hosts, and human-to-water contact. Because of their medical and epidemiological importance as intermediate hosts for *Schistosoma* parasites, freshwater snails of the *Biomphalaria* genus have garnered considerable research attention. Given the limited options for treating *S. mansoni* infections, a better understanding of the immunobiological interactions between the invertebrate host *Biomphalaria glabrata* and its parasite *S. mansoni* could be invaluable in developing new strategies for preventing and/or controlling Schistosomiasis diseases.

A number of studies published over the last two decades have contributed greatly to our understanding of *B. glabrata* innate immune mechanisms involved in the defense against pathogens. The discovery of recognition molecules such as lectins contributed to a better understanding of the mechanisms involved in pathogen recognition. Among this family of recognition molecules, the discovery of the somatically diversified FREPs (fibrinogen-related proteins) was an important advance in elucidating the immune-recognition step [3], [4]. Recently, FREPs were shown to play a crucial role in the fate of the interaction between *B. glabrata* and its trematode parasites [5]. A recent study described the putative involvement of the cytokine-like molecule, BgMIF (*B. glabrata* macrophage migration inhibitory factor) in the anti-parasite response of *B. glabrata* [6]. A number of studies have analyzed the response of *B. glabrata* to different immune challenges, allowing the identification of numerous putative immune genes that could play a key role in *B. glabrata* immune processes [7], [8], [9], [10], [11], [12]. Other studies based on comparisons of resistant and susceptible strains of *B. glabrata* to different trematode species from *Schistosoma* and *Echinostoma* genera [13], [14], [15], [16], [17] have also made a large contribution to the identification of factors putatively involved in the success or failure of parasite infection. Still other studies have explored mechanisms underlying compatibility polymorphism characteristics in certain *B. glabrata/S. mansoni* populations [18], [19], [20], [21]. These latter studies allowed the identification of two repertoires of polymorphic and/or diversified molecules that were shown to interact: the parasite antigens SmPoMucs (*S. mansoni* polymorphic mucins) and *B. glabrata* FREP immune receptors. The interaction profile of these molecules defines the compatible/incompatible status of a specific snail/schistosome combination (for a recent review, see [22]). Studies specifically dedicated to immune effectors have clearly demonstrated that *B. glabrata* production of reactive oxygen species (ROS), particularly H_2_O_2_, plays a crucial role in anti-schistosome defense [23], [24]. Moreover, hemocytes from *S. mansoni*-resistant snails have been shown to generate significantly more ROS than susceptible snails [25], [26], [27], and a reciprocal co-evolution has been demonstrated between ROS and ROS scavengers produced by sympatric populations of *B. glabrata* and *S. mansoni* [28]. Additional *B. glabrata* putative immune effectors have been identified, including LBP (lipopolysaccharide-binding protein) and BPI (bactericidal/permeability-increasing protein) [8], [29], and antimicrobial peptides [29], but their functions remain to be determined.

Using an interactome approach employing *B. glabrata* plasma and *S. mansoni* primary sporocyst extracts, we recently identified a new, putative cytolytic protein from *B. glabrata* that displays similarities to members of the β-PFT superfamily known to form channels in targeted membranes [30]. The most studied members of this superfamily are the aerolysin toxins secreted by several *Aeromonas spp.* [31], [32]. Other members of this β-PFT superfamily include the α-toxin produced by *Clostridium septicum* [33], the ε-toxin from *Clostridium perfringens* [34], the MTX (mosquito toxin)-type proteins secreted by *Bacillus sphaericus* [35], parasporin 2 and 4 from *Bacillus thuringiensis* [36], [37], monalysin from *Pseudomonas entomophila* [38], and the vibrioaerolysin of *Vibrio splendidus* [39]. Most of these proteins are produced by bacteria, as confirmed by a recent bioinformatic analysis of protein database entries displaying an aerolysin signature, which revealed that 70% of the putative β-PFTs identified came from bacteria [40]. These β-PFTs are most often produced by pathogenic bacteria. Several functional studies have clearly documented their virulence and mode of action (for a review, see [41], [42]). As an example, the entomopathogen *Pseudomonas entomophila* produces an aerolysin-like toxin that triggers the cytolysis and rupture of the drosophila intestinal epithelial barrier [38]. Other β-PFTs specifically target immune-competent cells, inducing their death [43], [44].

Some β-PFTs have also been identified in eukaryotic multicellular organisms, both animals and plants, but few have been characterized functionally. *Hydra viridissima* secretes different hydralysins that may be involved in protecting against predators or killing prey [45]. The seeds of *Enterolobium contortisiliquum* produce enterolobin, a pro-inflammatory protein that may protect against herbivore grazing [46], [47]. In cases in which the function of these eukaryotic β-PFTs was investigated, they were shown to share the same mode of action as their prokaryotic counterparts [48]. These β-PFTs, which are secreted as a soluble, inactive precursor called a protoxin, bind with high affinity to the glycosyl anchor of glycosylphosphatidyl inositol (GPI)-anchored proteins located on the surface membrane of target cells [49]. Some, including the aerolysins, show an affinity for carbohydrates, whereas others such as clostridium α-toxin lack this property [50], [51], [52]. This property of aerolysins is linked to their bilobal shape (for a review, see [42]): the large lobe common to all β-PFTs is involved in either oligomerization or binding to a GPI-anchored receptor, and the second smaller lobe contains a carbohydrate-binding domain. After binding to their ligand, all β-PFT protoxins oligomerize to form a ring-shaped heptameric channel [53], [54], [55]. Subsequent formation of a pore in the membrane requires an extracellular processing step that removes about forty amino acids of the aerolysin C-terminal region [56]. This last activation step can be achieved by pathogen proteases as well as by proteases from the host [38], [49], [57], [58].

Here, we report the cloning and characterization of a new β-PFT, which we have named Biomphalysin because it is produced by the *Biomphalaria* species, *B. glabrata*. This protein is the first cytolytic β-PFT protein from a mollusk to be characterized.

## Methods

### Ethics statement

Our laboratory holds permit #A66040 for experiments on animals from both the French Ministry of Agriculture and Fisheries, and the French Ministry of National Education, Research, and Technology. The housing, breeding, and care of animals utilized here followed the ethical requirements of our country. The experimenter also possesses an official certificate for animal experimentation from both French ministries (Decree #87–848, October 19, 1987). Animal experimentation followed the guidelines of the CNRS (Centre National de la Recherche Scientifique). The protocols used in this study have been approved by the French veterinary agency from the DRAAF Languedoc-Roussillon (Direction Régionale de l'Alimentation, de l'Agriculture et de la Forêt), Montpellier, France (Authorization #007083).

### Biological material and parasite challenge

*B. glabrata* and *S. mansoni* originated from Brazil and have been maintained in the laboratory for several years [59]. The parasite strain was maintained in hamsters (*Mesocricetus auratus*), as described previously [60]. Parasite recovery was conducted as follows: Rodent livers were collected in sterile saline solution (150 mM NaCl) containing an antibiotic/antimycotic mixture (penicillin 100 units/ml, streptomycin 0.1 mg/ml, amphotericin B 0.25 µg/ml; Sigma). After grinding, parasite eggs were filtered and washed. Miracidia were hatched from eggs in sterile water and concentrated by sedimentation on ice for 1 h. Primary sporocysts for tests of Biomphalysin antischistosomal activity were obtained by transferring miracidia to Chernin's balanced salt solution (CBSS) and maintaining at 26°C under normoxic conditions for 24 h [61]. The Bge cell line (ATCC, CRL 1494), derived from *B. glabrata*, was grown at 26°C under normoxic conditions in complete Bge medium, as described previously [62].

For parasite challenge, infestation experiments were performed on juvenile *B. glabrata* (5–6 mm in diameter). Snails were individually exposed to 10 miracidia of *S. mansoni* in 5 ml of pond water. The infectivity of the miracidia was confirmed by exposing additional snails to parasites at the same time and dissecting them at 15 d post-exposure. All groups were treated in the same manner and at the same time. Seven snails were collected at each point in challenge and control kinetics experiments. All experiments reported in the present study were repeated at least two times.

### 5′ and 3′ rapid amplification of cDNA ends (RACE)

Total RNA was extracted from a pool of ten snails using TRIzol reagent according to the manufacturer's instructions (Invitrogen). Full-length Biomphalysin cDNA was obtained by performing 5′ and 3′ RACE polymerase chain reactions (PCRs) using the GeneRacer kit, as described by the manufacturer (Invitrogen). Briefly, 5 µg of total RNA were treated with calf intestinal phosphatase to remove the 5′ phosphates. After phenol extraction, dephosphorylated RNA was decapped with a tobacco acid pyrophosphatase treatment. The GeneRacer RNA oligo provided by the manufacturer was ligated to the 5′end of the mRNA using T4 RNA ligase. Reverse transcription was performed using SuperScript III reverse transcriptase and GeneRacer Oligo dT primer. Primers for 5′ (5′-GGC TGG CTT AGT GCA TCT TGC GCT CT-3′) and 3′ (5′-GCT GTC AAC GAT ACG CTA CGT AAC G-3′) RACE were designed from a contig obtained by the assembly of *B. glabrata* expressed sequence tags (ESTs) identified by MASCOT analysis [30]. The amplification cycling conditions for both consisted of an initial denaturation step at 95°C for 5 min followed by 35 cycles of 30 s denaturation at 95°C, 30 s annealing at 55°C and 5 min extension at 68°C, followed by a final extension at 68°C for 10 min. Advantage 2 PCR enzyme (Clontech) was used in PCR reactions. RACE PCR products were analyzed by agarose gel electrophoresis and cloned into the pCR4-TOPO vector according to the manufacturer's instructions (Invitrogen). Clones were then sequenced using GATC facilities (GATC Biotech, Germany).

### Semi-quantitative RT-PCR

The tissue distribution of Biomphalysin mRNA was analyzed by preparing samples of albumen gland, head-foot, hepato-pancreas, and ovotestis, collected from ten snails under a binocular dissection microscope. Hemocytes from fifty snails were also collected from hemolymph after centrifugation at 10000×g for 10 min at 4°C. Total RNA from these different tissues, hemocytes, and Bge cells was extracted using TRIzol Reagent solution (Invitrogen) according to the manufacturer's protocol. Then, total RNA (10 µg) was treated with rDnase I (1 U/µg DNA; Ambion) to remove contaminating genomic DNA. cDNA was synthesized from DNA-free RNA (1 µg) by reverse transcription (RT) using random hexamer primers and Revertaid H minus M-MuLV reverse transcriptase (Fermentas). The absence of contaminating genomic DNA was confirmed using PCR primers (forward: 5′-CCC ATC TAT TGT TGG CAG ACC-3′, reverse: 5′-GTT TAG AGG TGC CTC TGT GAG-3′) for the actin gene (accession number U53348.1) designed to anneal to two different exons. Biomphalysin gene expression analyses were performed using primers allowing full-length cDNA amplification (forward: 5′-GGC TTA TAT TGC AGA AAA TGT TTT TA-3′, reverse: 5′-CTC TGA CAC AAT CAA GAC AAC AAG-3′). Semi-quantitative RT-PCR conditions were 94°C for 5 min followed by 32 cycles of 94°C for 30 s, 50°C for 30 s, 72°C for 1 min 30 s, and a final 5-min extension step at 72°C. PCR products were separated by electrophoresis on 2% agarose gels and cloned into pCR4-TOPO for subsequent sequencing.

### Real-time quantitative PCR

Total RNA was extracted either from a pool of seven snails or from Bge cells using TRIzol reagent. Reverse transcription was performed as described previously [21]. Real-time quantitative PCR (Q-PCR) was performed on cDNAs (diluted 50-fold with nuclease-free water) using the Light Cycler System 480 (Roche, Idaho Technologies). Sequences of primers used for amplification of Biomphalysin (forward: 5′-CTG ATT ACA CCT GGG C-3′, reverse: 5′-ACC CTT TCG TCC CAT AC-3′) and ribosomal protein S19 (forward: 5′TTC TGT TGC TCG CCA C, reverse: 5′-CCT GTA TTT GCA TCC TGT T-3′) were designed using LightCycler Probe Design software version 1.0 (Roche Diagnostics). Q-PCR reactions were performed according to the Light Cycler procedure. Amplification conditions were as follows: 20 s denaturation at 95°C followed by 40 cycles of 5 s at 95°C, 7 s at 60°C, and 12 s at 72°C. After amplification, the specificity of PCR products was determined by analyzing melting curves, acquired by heating the product at 95°C, cooling at 70°C for 20 s, and then slowly remelting (0.31°C/s) up to 95°C. Each PCR product was verified by sequencing. The results of melting curve analyses were quantified by determining the crossing-point value (Cp) using the second derivative maximum method of the Light Cycler Software 3.3 (Roche Diagnostics). Expression data were normalized to ribosomal protein S19 (accession number CK988928) levels [29]. The Biomphalysin-to-S19 transcription ratio was calculated using the relative quantification analysis module of the LightCycler 480 software. All Q-PCR experiments reported in the present study were repeated at least three times.

### Recombinant Biomphalysin production

A Biomphalysin cDNA lacking nucleotides encoding the signal peptide was amplified from the full-length gene by PCR using the primers, 5′-CGC TTA ATT AAA CAT ATG ACC CAA TGC ACC TAT TCC-3′ (forward) and 5′-TTA GTT AGT TAC CGG ATC CCT TAC TAG ACT TTC ACT TC-3′ (reverse), and subsequently cloned into the RTS pIVEX 1.4 Wheat Germ His_6_-tag Vector (5 Prime) using the In-Fusion HD Cloning Kit (Clontech), as described by the manufacturer. The expression vector was transformed into Stellar competent cells (Clontech), amplified by bacterial culture, and then purified using the Qiagen Plasmid Plus kit. The Biomphalysin protein was expressed *in vitro* as an N-terminal His_6_-tagged protein using the Rapid Translation System (RTS; 5 Prime), according the manufacturer's instructions. Briefly, 60 µg of expression vector were used per reaction of the RTS 500 Wheat Germ CECF Kit. The reaction was performed in the RTS ProteoMaster instrument by incubating at a temperature of 24°C for 2 h with shaking (900 rpm); the yield of N-terminal His_6_-tagged Biomphalysin was approximately 100 µg/reaction. Recombinant protein production efficiency was evaluated by Western blot analysis. Total protein extract was separated by sodium dodecyl sulfate-polyacrylamide gel electrophoresis (SDS-PAGE) on 12% gels and electrophoretically transferred onto a nitrocellulose membrane. The membrane was blocked by incubating for 3 h at room temperature in 4% non-fat dried milk in TBS (Tris-buffered saline)/0.05% Tween. The blot was subsequently incubated overnight at 4°C with anti-His antibody (Invitrogen) diluted 1∶5000 in 4% non-fat dried milk in TBS/0.05% Tween-20. The blot was washed three times with TBS/0.05% Tween-20 and incubated at room temperature for 1.5 h with anti-mouse antibody (diluted 1∶5000 in 1× phosphate-buffered saline [PBS], 4% milk, and 0.05% Tween-20), washed again with TBS/0.05% Tween-20, and then developed by incubating with an enhanced chemiluminescence (ECL) substrate (Pierce) followed by autoradiography. In order to determine rBiomphalysin concentration, several volumes (0.5 µl, 1 µl and 5 µl) of wheat germ extracts (WGE) containing or not rBiomphalysin were run on 12% SDS-PAGE, with a range of known amounts of bovine serum albumin (BSA, from 50 ng to 600 ng). The gel was subsequently stained with Coomassie blue using standard protocols [63]. rBiomphalysin concentration was assessed after separation and staining by densitometry analysis of the corresponding band. Briefly, the gel was scanned and digitized using a GS 800 calibrated densitometer (Bio Rad). rBiomphalysin and BSA colorations were estimated using the software Quantity One 1-D Analysis Software 4.6. As Coomassie blue only colors aromatic and basic amino acids which number vary between proteins, we verified the amount of basic and/or aromatic residues in both compared proteins. BSA and rBiomphalysin contain 598 and 607 amino acids and they display 157 and 154 basic and/or aromatic residues, respectively. Among them, BSA contains 17% of basic residues and 12% of aromatic residues and rBiomphalysin contains 13% of basic residues and 13% of aromatic residues. These close values allowed us to estimate the concentration of rBiomphalysin in WGE using the standard curve obtained with the different BSA amounts. This estimation is 90 ng of rBiomphalysin per µl of WGE.

### Hemolytic assay

Hemolytic assays were performed according to a previously described procedure [64]. Briefly, different amounts of crude recombinant Biomphalysin (0.61–500 nM; total volume, 20 µl) were mixed with a 3% suspension of sheep erythrocytes (120 µl) and *B. glabrata* plasma or PBS (40 µl), and incubated overnight at 37°C with gentle agitation. After a 2-min centrifugation at 500×g, the supernatants were collected and their absorbance was measured at 405 nm (AD 340; Beckman Coulter). Percent hemolysis was calculated according to the equation, 100×(A_405_ sample−A_405_ negative control)/(A_405_ positive control−A_405_ negative control), where negative control corresponds to the same amount of RTS 500 reaction performed using empty pIVEX 1.4 vector (i.e. without the Biomphalysin gene) and positive control corresponds to total lysis caused by a nonionic surfactant (20 µl of 10% Triton X-100 in PBS in place of recombinant protein).

### Cytotoxicity of Biomphalysin toward *S. mansoni* sporocysts

Primary sporocysts (Sp1) were obtained by *in vitro* transformation of 400 miracidia and then were exposed to rBiomphalysin (30 nM) with or without ultracentrifuged *B. glabrata* plasma (40 µl in a 200 µl reaction volume). Ultracentrifugation step (30,000 rpm, 3 h, 4°C) is used to remove free haemoglobin from plasma. Cytotoxicity was determined by direct light microscopic observations of (i) vacuolization, (ii) focal *lysis* of the *tegumental* matrix and/or underlying muscle fibers, and (iii) mortality. Primary sporocysts were considered dead if they failed to exhibit motility and/or beating of flame-cell flagella. Experiments were conducted in duplicate on 12-well plates using 25 sporocysts per well. Kaplan-Meier survival analyses followed by pairwise log-rank tests were used to compare survival data, as described previously [65].

### Immunocytochemical detection of Biomphalysin on *S. mansoni* sporocysts

One hundred sporocysts were incubated with 40 µl of WGE with rBiomphalysin protein (∼60 nM) or WGE for 1 h in the presence or absence of *B. glabrata* plasma. Then, sporocysts were rinsed and fixed by incubating with 4% paraformaldehyde in PBS for 1 h at room temperature. Afterwards, parasites were rinsed with PBS and centrifuged (1 min, 800×g) onto poly-D-Lysine–coated slides (CultureSlides; BD Falcon). Slides were blocked by incubating with PBS containing 3% bovine serum albumen (BSA) for 2 h at room temperature. Parasites were then incubated for 90 min with an anti-His antibody diluted 1∶500 (Life Technologies). After being washed three times in PBS, parasites were incubated with an Alexa Flour 594-conjugated anti-mouse IgG (Life Technologies) diluted 1∶1000 in PBS/1% BSA for 45 min at room temperature. After rinsing, slides were mounted in Dako fluorescent mounting medium (Dako) and examined using a fluorescence confocal laser-scanning microscope (Zeiss LSM 700, Tecnoviv platform). All images were acquired under the same conditions (63×, 1.40 oil DIC M27, pinhole 1.00 [arbitrary units], 8-bit sampling, average of 4 frames at 1400×1400 pixels). The resolution obtained was 13,778 pixels/µm. For presentation, images were imported into ImageJ software.

### Detection of Biomphalysin on *S. mansoni* sporocysts by *in vitro* binding assay

Fifty sporocysts were recovered from cell culture plates after a 6-h incubation at 27°C in the presence or absence of *B. glabrata* plasma, with or without crude recombinant protein at the same concentration used for immunocytochemistry. Sporocysts were centrifuged for 5 min at 600×g and washed three times with CBSS medium. The recovered sporocyst pellets were subsequently denaturated by incubating for 10 min at 80°C in Laemmli buffer (Laemmli, 1970). The entire sample was resolved by SDS-PAGE on a 10% gel and treated as described previously.

### Bioinformatic analysis

The presence of a signal peptide was determined by a primary structure analysis performed using SignalP 3.0. Potential glycosylation sites were predicted using NetNglyc 1.0 and NetOGlyc 3.1 (http://www.cbs.dtu.dk/services/). Protein domain searches were performed using SMART (http://smart.embl-heidelberg.de/) and Motif Scan (http://hits.isb-sib.ch/cgi-bin/PFSCAN) software. α-helical and β-sheet regions were identified by secondary structure prediction using Jpred3 and HHpred servers (http://toolkit.tuebingen.mpg.de/hhpred). Sequences were searched for putative transmembrane domains (TMDs) using the PRED-TMBB server [66], [67].

Prediction of Biomphalysin three-dimensional (3D) structure and alignment with the crystal structure of proaerolysin were performed using I Tasser and TM-align servers [68], [69]. The 3D structure was obtained by multiple threading using the I-Tasser server (available online), which combines two protein structure prediction methods: threading and *ab initio* prediction [70]. The quality of the computed model was estimated by the C-score (Confidence score). C-scores are typically in the range of −5 to 2, where a high C-score signifies a model with a high confidence; models with a C-score greater than −1.5 are predictive of correct folding. We obtained a C-score of −0.5 for Biomphalysin protein and 1.5 for its aerolysin domain, values that satisfy this acceptability criterion. Structural similarities between the functional domain of aerolysin and Biomphalysin were determined by calculating a TM-score. A TM-score greater than 0.5 reveals significant alignment, whereas a TM-score less than 0.17 indicates random similarity.

### Phylogenetic analysis

To investigate the phylogenetic position of Biomphalysin, we retrieved sequences of aerolysin homologues from a recent study [71]. Forty-seven sequences (Table 1) from organisms belonging to animal, plant, fungi, and bacterial kingdoms were used to construct a phylogenetic tree. Selected sequences were then aligned using the MUSCLE algorithm implemented in CLC Sequence DNA Workbench 6.6.2 software (CLC bio). Poorly aligned regions were trimmed using trimAl v1.4 with *automated1* option [72]. Phylogenetic analyses were performed using Bayesian and maximum-likelihood (ML) inferences. ProtTest v3.2 was used to select the model of protein evolution (amino acids substitution) that best fit the multiple sequence alignment [73]. The WAG+F model was selected. A Bayesian analysis was performed using MrBayes 3.2.1 [74] with 1,500,000 generations. We estimated that the analysis reached convergence when the average standard deviation of split frequencies between the two runs was less than 0.01 and the potential scale reduction factor reached 1.0 (burn-in = 3750). The robustness of the nodes was evaluated using the Bayesian posterior probabilities. A maximum likelihood analysis was also performed on the same alignment using PhyML 3.0 [75]. The reliability of the nodes was tested using a bootstrap test (100 replicates). Finally, the tree was edited using FigTree v1.3.1 (http://tree.bio.ed.ac.uk).

**Table 1:** Sequences of Biomphalysin used to construct the phylogenetic tree.

Name	Organism	gi-Accession
Aerolysin	*Aeromonas hydrophila*	113485
Aerolysin precursor	*Aeromonas salmonicida*	2501303
hemolysin	*Aeromonas sobria*	148751473
Biomphalysin	*Biomphalaria glabrata*	KC012466
Hydralysin-2	*Bacillus thuringiensis* IBL 200	228911714
Hydralysin-2	*Bacillus thuringiensis serovar berliner*	228943424
Crystal protein	*Bacillus thuringiensis*	51090285
Alpha-toxin	*Clostridium botulinum*	253771270
Hypothetical protein CC1G_11805	*Coprinopsis cinerea*	299752492
Hypothetical protein CC1G_08369	*Coprinopsis cinerea*	299745505
Hypothetical protein CC1G_10318	*Coprinopsis cinerea*	299746325
Epsilon toxin precursor	*Clostridium perfringens*	315320199
Alpha-toxin	*Clostridium septicum*	27531080
Cytolytic protein enterolobin	*Enterolobium contortisiliquum*	2501305
hypothetical protein HCH_03563	*Hahella chejuensis*	83646295
hypothetical protein	*Hydra magnipapillata*	221130489
Predicted : similar to hydralysin	*Hydra magnipapillata*	221104132
Hydralysin-1	*Hydra viridissima*	39931521
Hydralysin-2	*Hydra viridissima*	74997549
Hydralysin	*Hydra vulgaris*	74996299
Secreted salivary gland peptide	*Ixodes scapularis*	241568118
Hypothetical protein IscW_ISCW013639	*Ixodes scapularis*	241838790
Pore-Forming Lectin	*Laetiporus sulphureus*	61680142
Hemolytic lectin LSLb	*Laetiporus sulphureus*	32261218
Hemolytic lectin LSLc	*Laetiporus sulphureus*	32261220
predicted protein	*Nematostella vectensis*	156373767
Predicted protein	*Nematostella vectensis*	156403083
Predicted protein	*Nematostella vectensis*	156403081
Cytotoxin	*Pseudomonas* phage phiCTX	17313218
Hypothetical protein PBPRB1941	*Photobacterium profundum*	54303595
Hypothetical protein Pecwa_1694	*Pectobacterium wasabiae*	261820982
Conserved hypothetical protein	*Ricinus communis*	255557038
Conserved hypothetical protein	*Ricinus communis*	255557040
Conserved hypothetical protein	*Ricinus communis*	255565071
Aerolysin/hemolysin/leukocidin toxin	*Shewanella baltica*	153000088
Pore-forming toxin-like protein Hfr-2	*Triticum aestivum*	57233444
hypothetical protein VIBC2010_03220	*Vibrio caribbenthicus*	312885265
Hypothetical protein VIBC2010_07664	*Vibrio caribbenthicus*	312883148
Hypothetical protein VIC_000420	*Vibrio coralliilyticus*	260775048
Pre-provibrioaerolysin	*Vibrio harveyi*	153831849
Hypothetical protein VINI7043_24062	*Vibrio nigripulchritudo*	343495132
hypothetical protein V12B01_24484	*Vibrio splendidus*	84387032
Hypothetical protein VITISV_020655	*Vitis vinifera*	147838248
Uncharacterized protein LOC100256767	*Vitis vinifera*	225465417
Uncharacterized protein LOC100251726	*Vitis vinifera*	225465423
Uncharacterized protein LOC100194135	*Zea mays*	212722702
Uncharacterized protein LOC100275466	*Zea mays*	226531001

### Accession number

Nucleotide sequence data reported in this paper are available in the GenBank database under the accession number KC012466

## Results

### Molecular characterization of Biomphalysin

**Figure 1:**
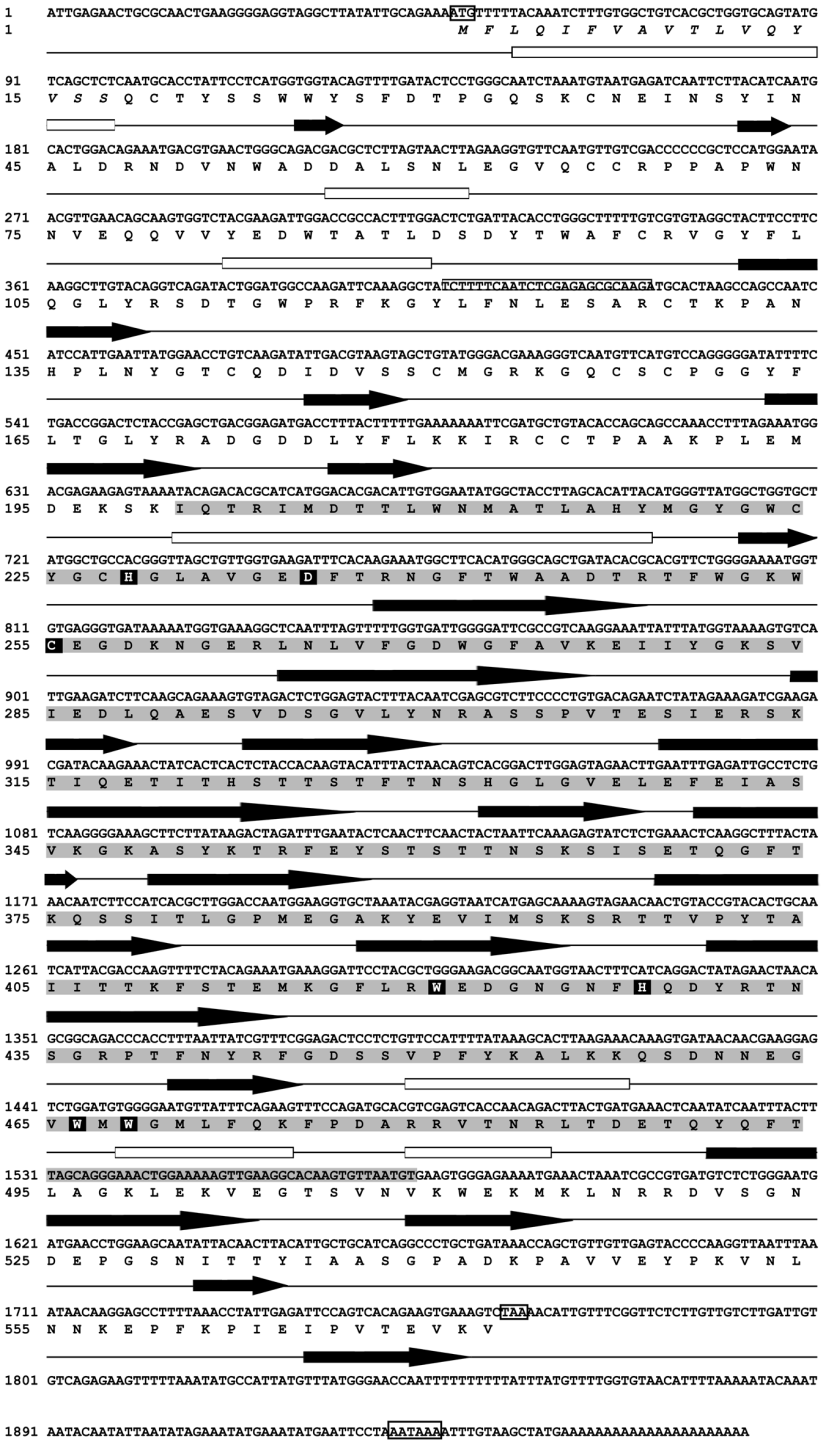
Full-length cDNA sequence and predicted amino acid sequence of Biomphalysin. The nucleotide sequence of Biomphalysin is shown with the initiation codon (ATG), terminator codon (TAA), and polyadenylation sites (AATAAA) boxed. For the protein sequence, italics are used to denote the putative signal peptide and the grey shadow region indicates the aerolysin domain signature. Amino acid residues crucial for oligomerization and those involved in cytolytic activity are in white on a black background. The positions of secondary structure elements were predicted using the Jpred3 server; α-helices are indicated by white rectangles and β-strands are depicted as black arrows.

**Figure 2:**
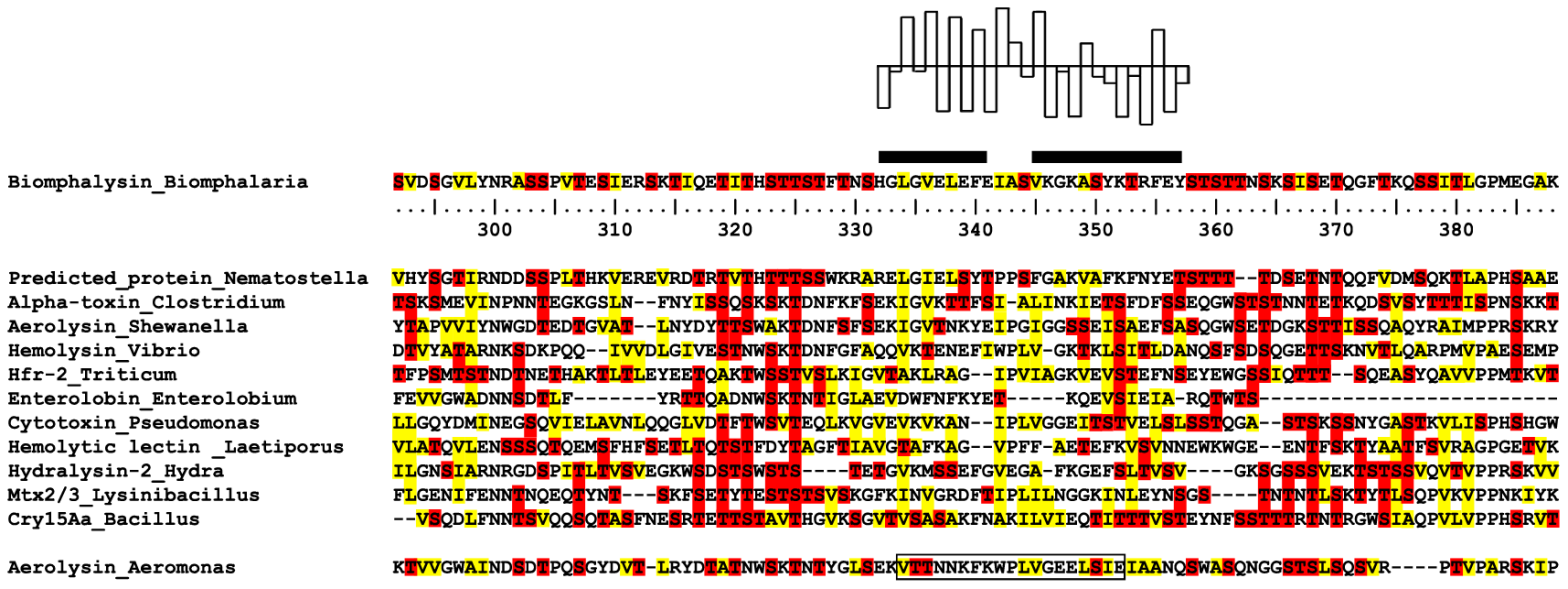
Identification of the Biomphalysin TMD, a conserved feature of the β-PFT family. Multiple alignments of aerolysin-like proteins were performed using the HHpred server. The UniProt accession numbers of the selected β-pore forming toxins are as follows: *N. vectensis*, A7RL10; *Clostridium botulinum*, C6DXY7; *Shewanella baltica*, A3D2W6; *Vibrio sp.*, A8T2E9; *A. hydrophila*, Q8RN77; *Triticum aestivum*, Q4JEV5; *E. contortisiliquum*, P81007; *Pseudomonas aeruginosa*, P14608; *Laetiporus sulphureus*, Q7Z8V0; *H. viridissima*, Q564A5; *Lysinibacillus sphaericus*, Q45471; *B. thuringiensis*, Q45729. The TMD, outlined in black, was predicted using the PRED-TMBB server (PREDiction of TransMembrane Beta Barrels proteins). The TMD is flanked by two hydrophilic regions; serine and threonine residues in these regions are shown in red. Hydrophobic residues (valine, leucine, isoleucine, and alanine) are shown in yellow. The putative TMD defined according to [40] is boxed. A hydropathy plot of the predicted TMD, according the hydrophobicity scale of Kyte and Doolittle [85], is indicated above the amino acid sequence.

**Figure 3:**
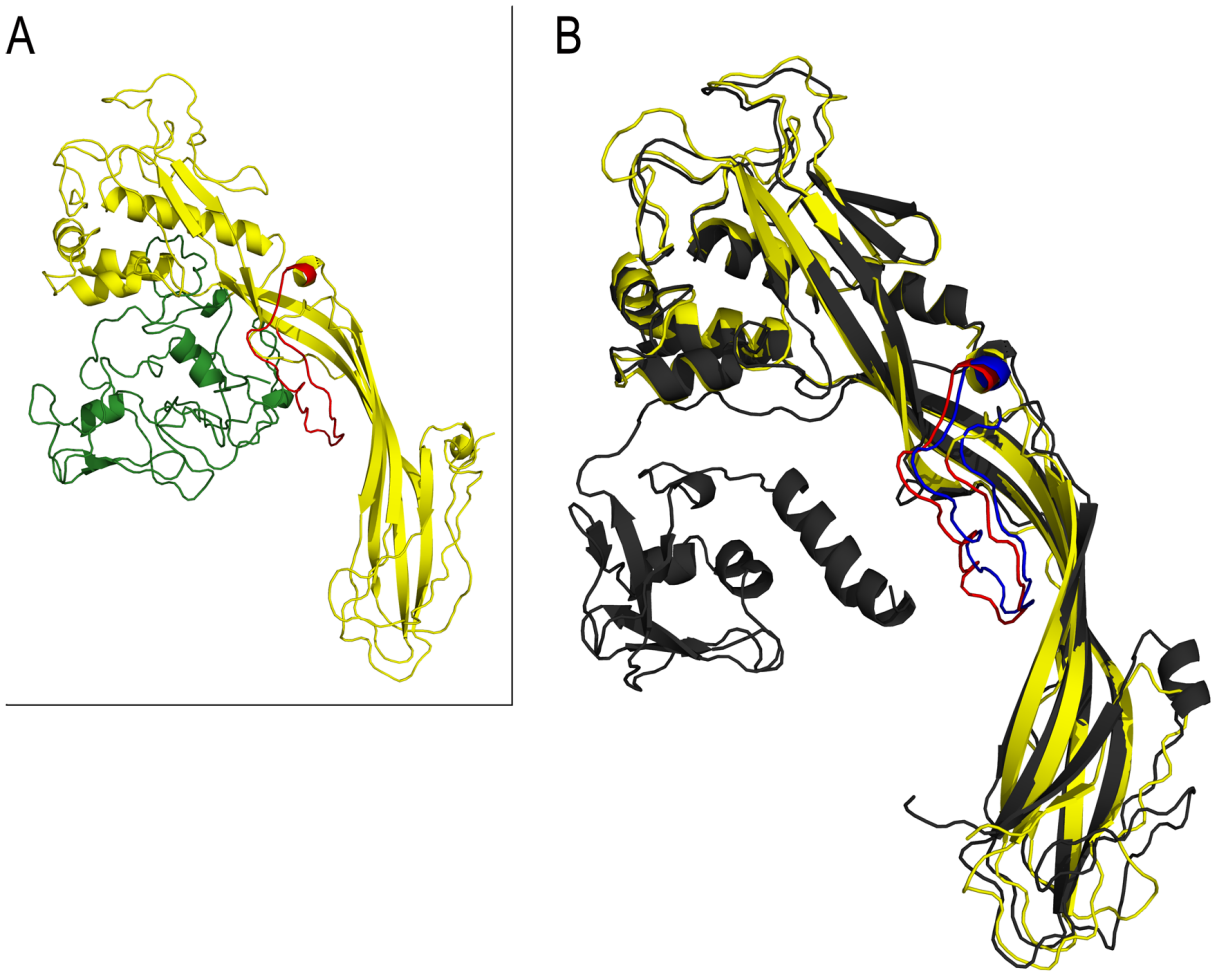
Structural alignment between the aerolysin domain of Biomphalysin and proaerolysin protein. A. Biomphalysin structure prediction. Biomphalysin and its aerolysin domain 3D structures were predicted by the I Tasser server. The quality of both predictions was estimated by calculating C-scores for the 3D structures of Biomphalysin (−0.5) and the aerolysin domain (1.5). The Biomphalysin protein is composed of two lobes: a small lobe (green) and a large lobe (yellow). The TMD predicted by PREDD TMBB software is shown in red. B. Structural comparison of the aerolysin domain of Biomphalysin (yellow) and the crystal structure of proaerolysin (PDB accession number 1Z52) template (grey). The proaerolysin TMD is shown in blue. The TMD predicted for the Biomphalysin is shown in red. A TM score of 0.92 was obtained over 372 aligned amino acids. All pictures were generated by PyMOLWin.

In a previous study designed to characterize the interactome between *B. glabrata* plasma and *S. mansoni* primary sporocyst extracts, we identified a partial coding sequence corresponding to a new, putative cytolytic protein from *B. glabrata* [30]. Because of its similarities to proteins of the β-PFTs superfamily, the corresponding protein was named Biomphalysin. In the present work, we obtained a full-length cDNA clone of Biomphalysin using the RACE method (Figure 1). The complete sequence is 1972 base pairs (bp) in length and displays a 5′-untranslated region (UTR) of 47 bp, a 3′-UTR of 206 bp, and an open reading frame (ORF) of 1719 bp (GenBank accession number : KC012466). The ORF encodes a precursor protein of 572 amino acid residues, of which the first 17 amino acids correspond to a putative signal peptide, as predicted by the SignalP program. After signal peptide removal, Biomphalysin displayed a theoretical pI of 6.2 and predicted molecular weight of 62.8 kDa. An analysis of putative post-translational modifications using the NetOglyc and NetNglyc server suggested the absence of O-glycosylation and a putative N-glycosylation event at N_530_. A BLASTP similarity analysis revealed significant similarities with members of the β-PFT superfamily, including aerolysin-like proteins. The most closely related sequence was a hypothetical protein from *Nematostella vectensis* (XP_001629482) with 55% similarity and 39% identity (E-value = 2×10^−123^). An analysis with the SMART and MotifScan programs revealed an aerolysin signature (pfam 01117) at residues 178–525 of Biomphalysin (E-value = 1.3×10^−43^). HHpred (homology detection and structure prediction by HMM-HMM comparison) software predicted a high structural homology with the pore-forming lobe of aerolysin (E-value = 1×10^−89^) and a high proportion of β sheets (31%). Members of the aerolysin-like protein family are virulence factors belonging to the superfamily of β pore-forming toxins produced and secreted predominantly by Gram-positive and -negative bacteria [40], [76]. They exert cytolytic activity triggered by channel formation in target cell membranes through insertion of β-hairpins, which form a β-barrel pore [48]. The formation of this pore requires a proteolytic cleavage event and the presence of the pore-forming transmembrane domain (TMD). Despite the poor level of similarity at the amino acid level, a putative TMD (from His_332_ to Tyr_357_) corresponding to an amphipathic sequence involved in the formation of membrane-inserted β barrels was clearly identified (Figure 2). This membrane-spanning domain was flanked by two hydrophilic regions, a feature shared by members of the aerolysin toxin family, like *cnidarian* hydralysins [45], *C. perfringens* α toxin [77], and *Aeromonas hydrophila* proaerolysin [55], [78]. A number of studies have identified several key amino acids that are involved in pore formation through oligomerization of β-PFT or that contribute to cytolytic activity. These critical residues are conserved in the Biomphalysin amino acid sequence, and include His_228_, Asp_235_ and Cystein_255_, which play a crucial role in oligomerization of the heptameric ring [79], [80], [81]; Tryp_466_ and Tryp_468_, which are involved in membrane penetration, as evidenced by reduced efficiency of pore formation in proteins mutated at these residues [82]; and Tryp_420_ and His_428_, which are involved in binding of the proaerolysin to its membrane receptor. In addition, as the proaerolysin cytolytic toxin, the Biomphalysin protein displayed two distinct lobes (Figure 3A). A 3D alignment of the aerolysin domain of Biomphalysin with the proaerolysin template was performed using I-Tasser and TM-align servers. This latter analysis revealed a high degree of similarity between the two structures (TM-score = 0.92; Figure 3B). Despite these structural similarities, neither C-type lectin motifs nor a cleavage site were found using motif prediction software [50], [83]. Biomphalysin displays a structural feature that distinguishes it from other β-PFTs. Indeed, Biomphalysin possesses a second lobe which displays no lectin-like domain as it has been reported for aerolysin [50].

**Figure 4:**
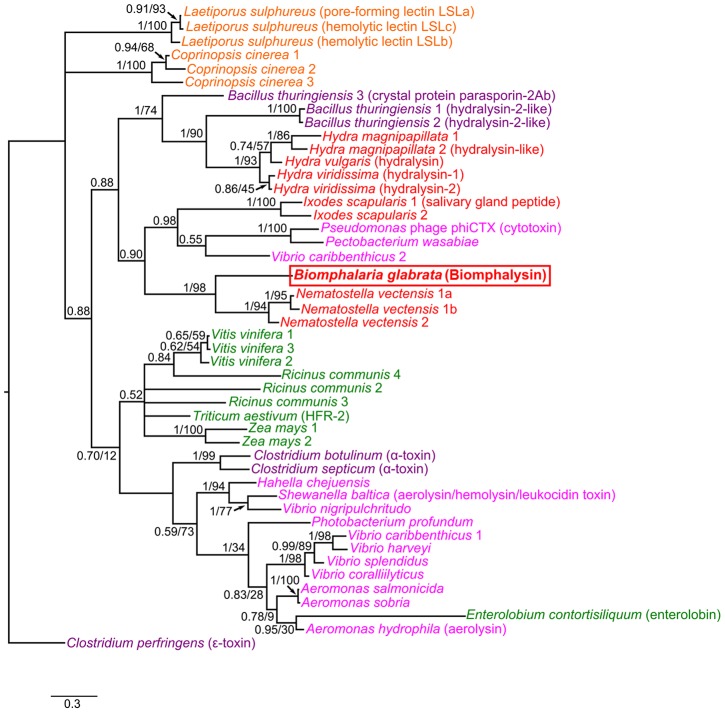
Phylogenetic tree of aerolysin-like molecules. The phylogenetic tree of aerolysin-like molecules is shown, with values of posterior probabilities and bootstraps for both Bayesian and ML analyses indicated at each node. Only the Bayesian tree is represented here, but both Bayesian and ML analyses produced trees with similar topologies. Aerolysin-like sequences from animals are represented in red, those from plant in green, those from fungi in dark red, those from Gammaproteobacteria in pink, and those from Firmicutes in purple. The sequence of ε-toxin from *C. perfringens* was used to root the tree. The scale bar corresponds to 0.3 estimated amino-acid substitutions per site.

A phylogenetic tree was subsequently constructed using 46 sequences of aerolysin-like toxins from different kingdoms (Table I). As expected, the Biomphalysin sequence in this tree appeared to be closely related to a predicted, uncharacterized protein identified in the cnidarian, *N. vectensis* (Figure 4). Curiously, and as also described by another phylogenetic study on β-PFTs [71], this tree comprises several monophyletic groups that contain both eumetazoa and bacteria. This taxonomic distribution suggests that the Biomphalysin gene was probably horizontally transferred several times from bacteria to eumetazoa.

Together, these data strongly suggest that Biomphalysin could be a cytolytic protein related to the β-PFT superfamily.

### Biomphalysin hemolytic activity

**Figure 5:**
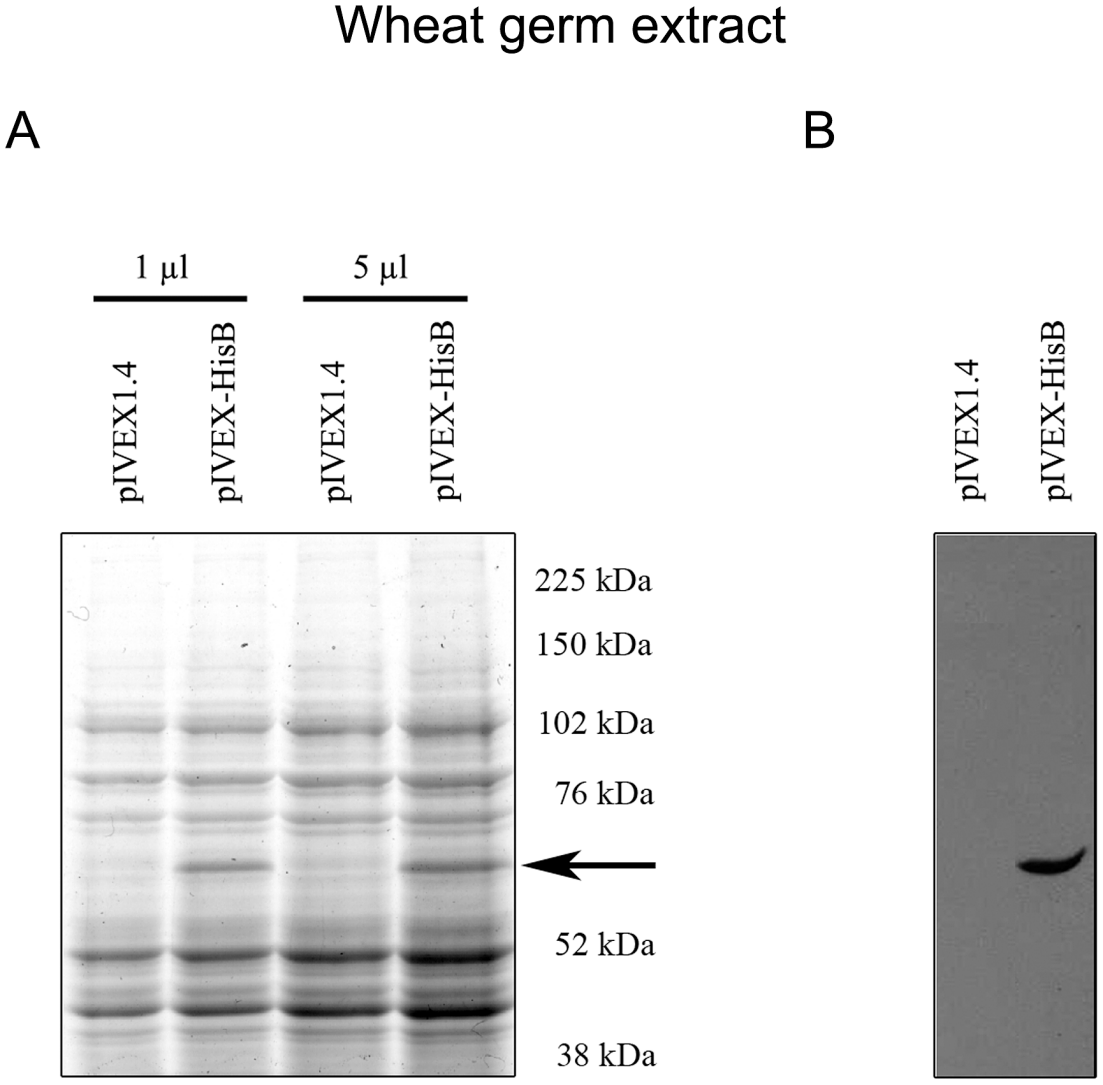
Analysis of rBiomphalysin expressed *in vitro* using wheat germ extracts (RTS 500). A. The amount of rBiomphalysin production was assessed by comparing the RTS 500 His-tagged Biomphalysin reaction (lanes 2 and 4) with 1 and 5 µl of RTS 500 control reactions (lanes 1 and 3) in Coomassie blue-stained, 12% SDS-PAGE gels. Values on the right indicate the masses of the molecular weight markers, whereas the arrow indicates the position of the His-tagged Biomphalysin band (lanes 2 and 4). B. Western blot analysis of RTS 500 control (lane 1) and Biomphalysin (lane 2) reactions. Five microliters of each reaction were separated by SDS-PAGE on a 12% gel and then electrotransferred onto a nitrocellulose membrane. The membrane was then incubated sequentially with a monoclonal anti-His_6_ antibody and a goat anti-mouse IgG, and immunoreactive proteins were visualized by ECL.

**Figure 6:**
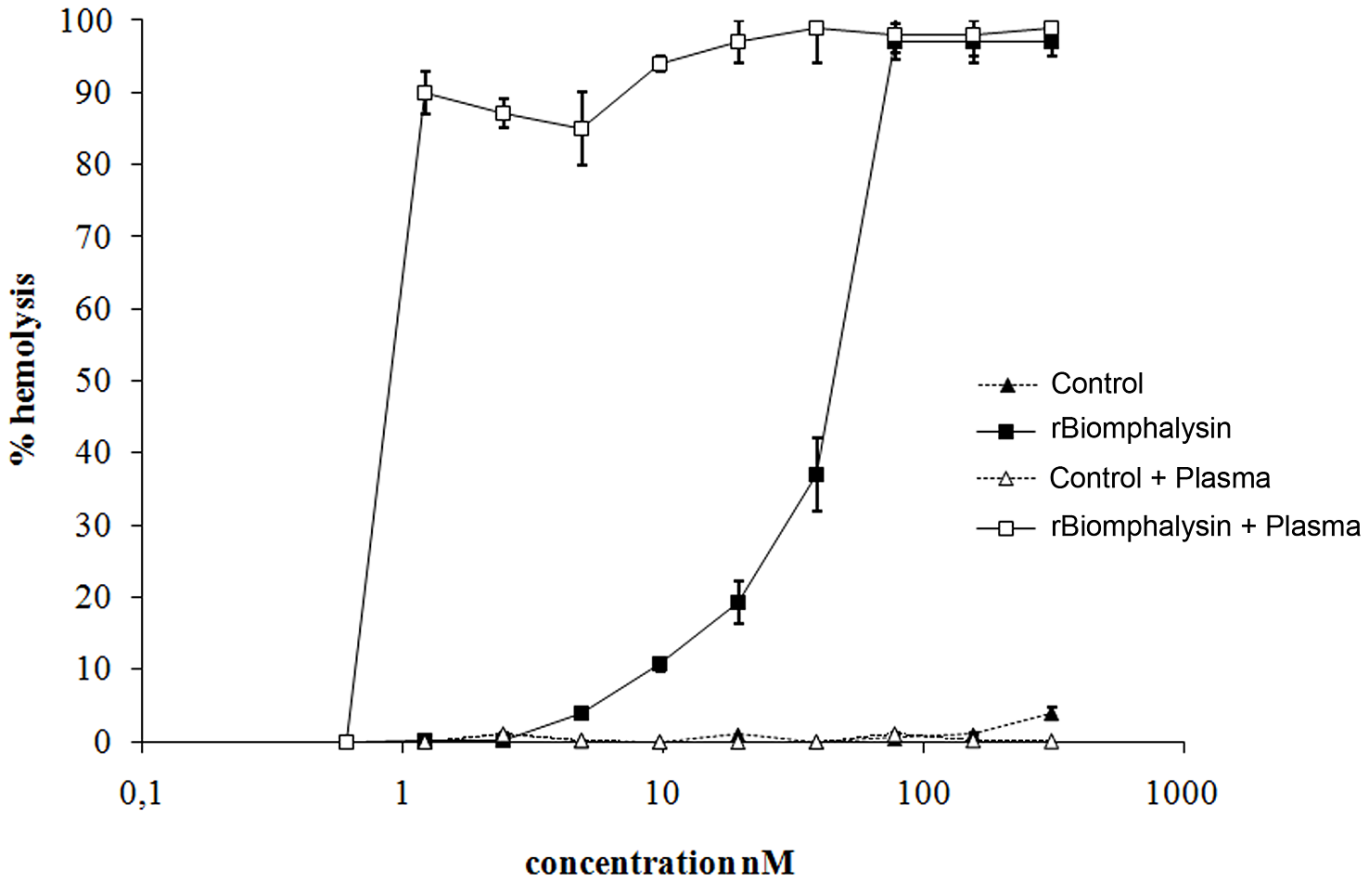
rBiomphalysin hemolytic activity. Different concentrations of rBiomphalysin, prepared by serial dilution of the Biomphalysin RTS 500 reaction, were tested for hemolytic activity towards sheep erythrocytes. The RTS 500 control reaction (WGE) was used as a negative control, and a 10% Triton-X100 solution was used as a positive control. The hemolytic activity of RTS 500 reactions was assessed in the presence or absence of *B. glabrata* plasma. The hemolytic activity of rBiomphalysin without and with plasma is shown by full and empty squares, respectively; full and empty triangles denote the corresponding activity for the negative control reaction without and with plasma. Hemolytic assay was performed with three replicates and error bars represent SD.

Most aerolysins characterized to date display potent hemolytic activity. In order to investigate the cytolytic capacity of Biomphalysin, we produced a recombinant protein flanked by an N-terminal hexa-histidine tag. We encountered some difficulties in producing recombinant Biomphalysin (rBiomphalysin) in our bacteria system. We tested different bacterial strains, including *Escherichia coli* BL21 (DE3); BL21 (DE3) pLysE; and BL21(DE3) CodonPlus, Rosetta (with and without classical chaperone expression.). With most of these systems, we obtained a low production level or a cleaved protein (data not shown). Consequently, we decided to produce the rBiomphalysin using an *in vitro* recombinant expression system based on wheat germ extract and cell free transcription and translation system (RTS). Expression of the rBiomphalysin was confirmed by Coomassie blue stained SDS-PAGE (Figure 5A) and by Western blot using an anti-His antibody (Figure 5B) that revealed a tagged protein with the expected size. Wheat germ extract containing rBiomphalysin and wheat germ extract alone used as negative control were tested for haemolytic activity toward sheep red blood cells in presence or absence of snail plasma. Hemolysis was observed for the WGE containing rBiomphalysin in presence or in absence of plasma (Figure 6). The rBiomphalysin concentration necessary for 50% lysis (Ha_50_) under plasma-free conditions was much higher (50 nM) than that required when rBiomphalysin (1 nM) was incubated with ultracentrifuged plasma, suggesting that a cofactor present in plasma enhanced the cytolytic effect of Biomphalysin.

### rBiomphalysin anti-schistosomal activity

**Figure 7:**
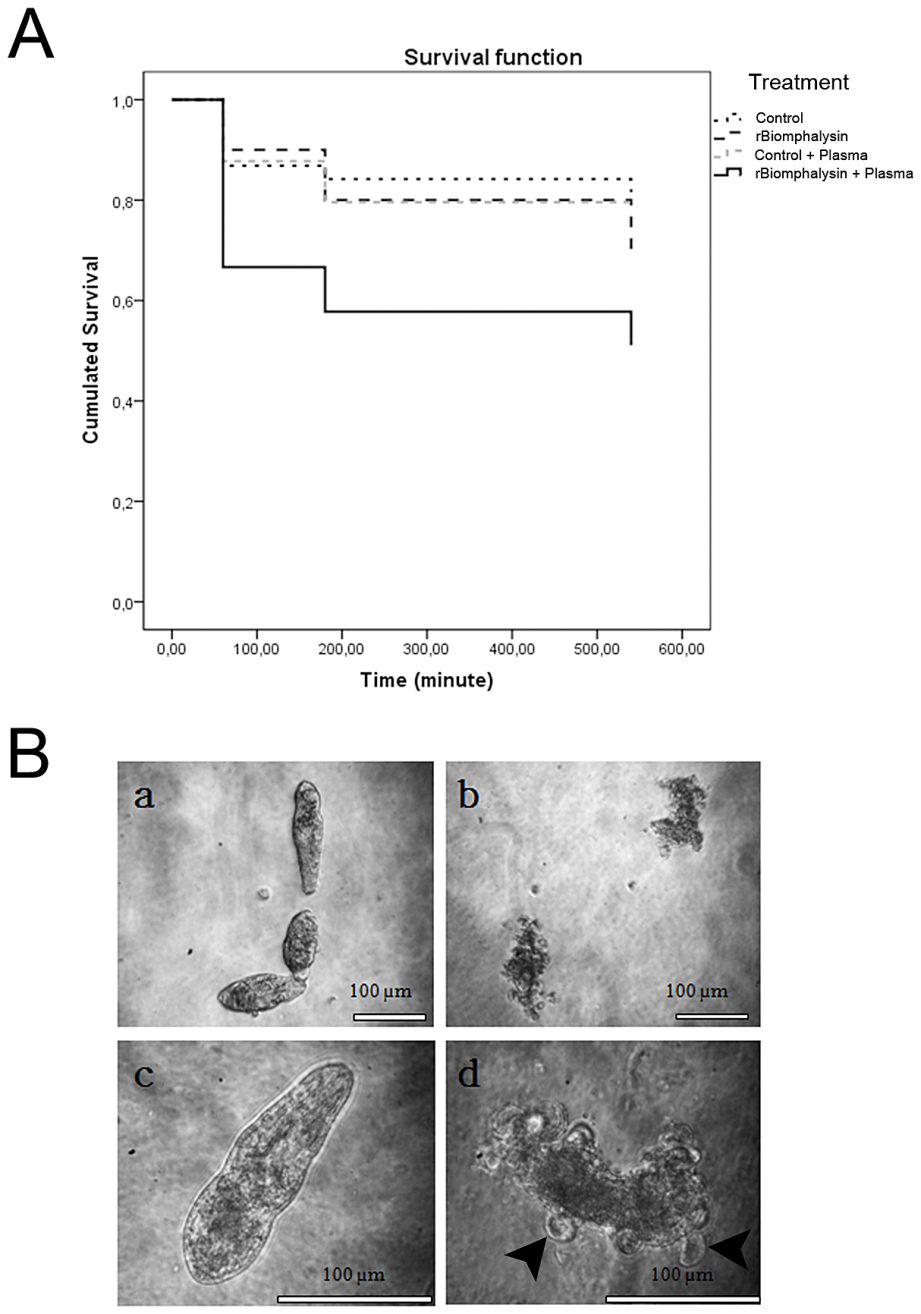
Cytolytic activity and *in vitro* effects of rBiomphalysin on *S. mansoni* sporocysts. A. Kaplan-Meier analysis of sporocyst treated with rBiomphalysin in the presence or absence of *B. glabrata* plasma. B. (a and c) Sporocysts treated with WGE (control). (b and d) Sporocysts treated with rBiomphalysin and plasma. Black arrows indicate swollen cells without cilia.

We next investigated Biomphalysin activity on intra molluscan stages of *S. mansoni* (primary sporocysts). To accomplish this, we treated an *in vitro* culture of primary sporocysts with 30 nM rBiomphalysin with or without *B. glabrata* plasma. Motility and beating of flame-cell flagella were assessed every hour during the first 9 h after initiating treatment to distinguish live and dead parasites. Exposure of sporocysts to *B. glabrata* plasma alone or WGE (without rBiomphalysin) or both produced no obvious morphological alterations. After 9 h of treatment with any combination of control conditions, a maximum of 20% of sporocysts died (Figure 7A). No differences were evident between larvae exposed to rBiomphalysin alone and those in various control groups. However, the combination of rBiomphalysin and *B. glabrata* plasma clearly enhanced parasite mortality. We found that parasites died faster: 35% (p<0.05, Fisher's exact test) were dead in less than 1 h; at the final time point, 50% of the parasites were dead (Figure 7A). The statistical significance of this result was investigated and confirmed using the Kaplan-Meier test (p = 0.004). A microscopic examination of the effects of rBiomphalysin plus *B. glabrata* plasma revealed severe tegumental alterations of *S. mansoni* sporocysts, with evidence of swollen cells sprouting from larvae, darkened and granular bodies and, ultimately, parasite disintegration (Figure 7B).

**Figure 8:**
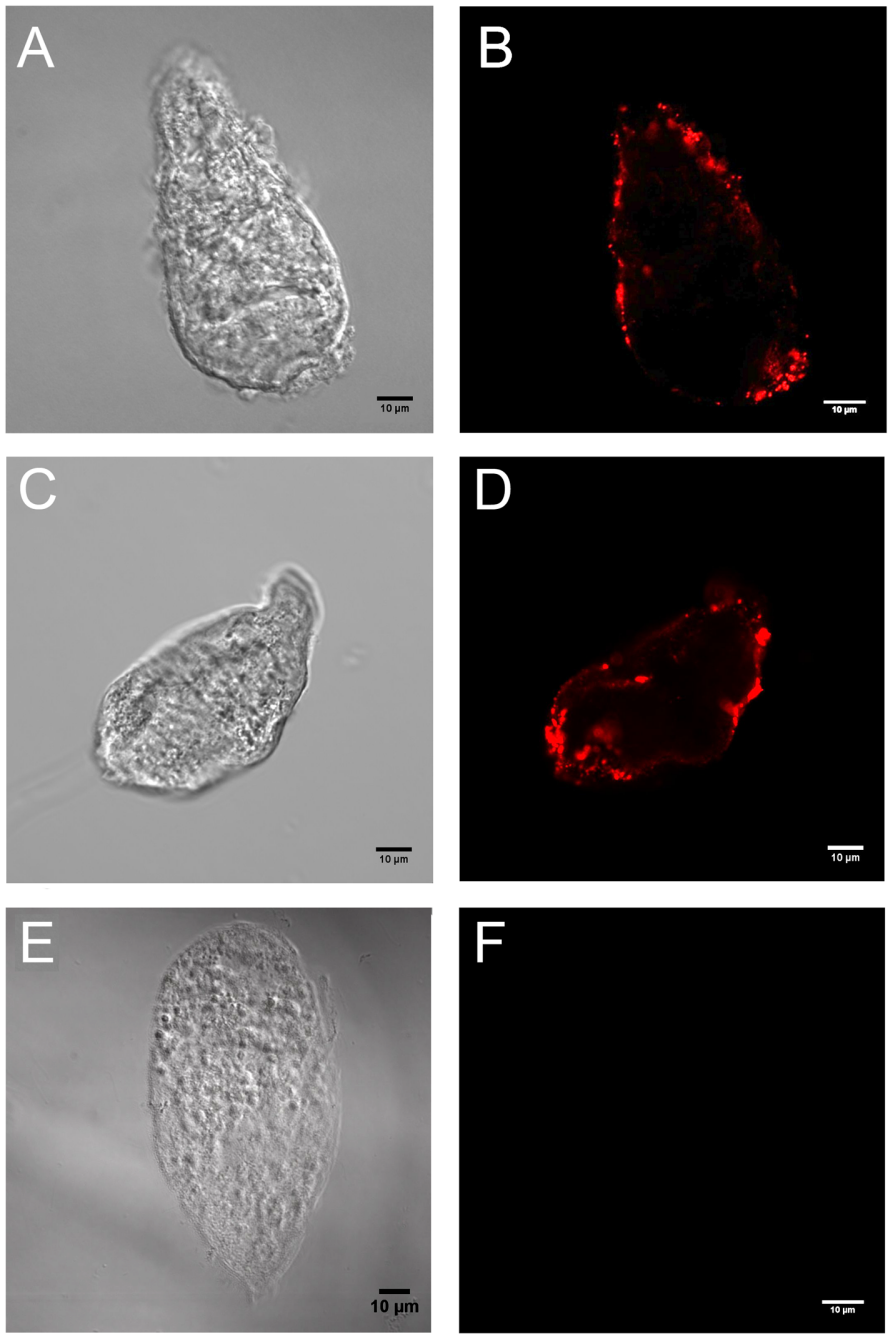
Immunolocalization of rBiomphalysin on *S. mansoni* sporocyst. Sporocysts were treated with rBiomphalysin in presence (A and B) or in absence (C and D) of snail plasma and immunostained using anti-His primary IgG and Alexa Fluor 594-conjugated secondary antibody. Binding of rBiomphalysin to sporocyst membranes was detected by aggregates formation on the parasite tegument in both conditions. Under the same image-acquisition conditions, no signal was detected for the negative control, consisting of incubation of sporocysts with plasma and wheat germ extract alone (E and F). A, C and E represent the image taken under Nomarski light microscopy, whereas B, D and F are the corresponding confocal fluorescent pictures.

**Figure 9:**
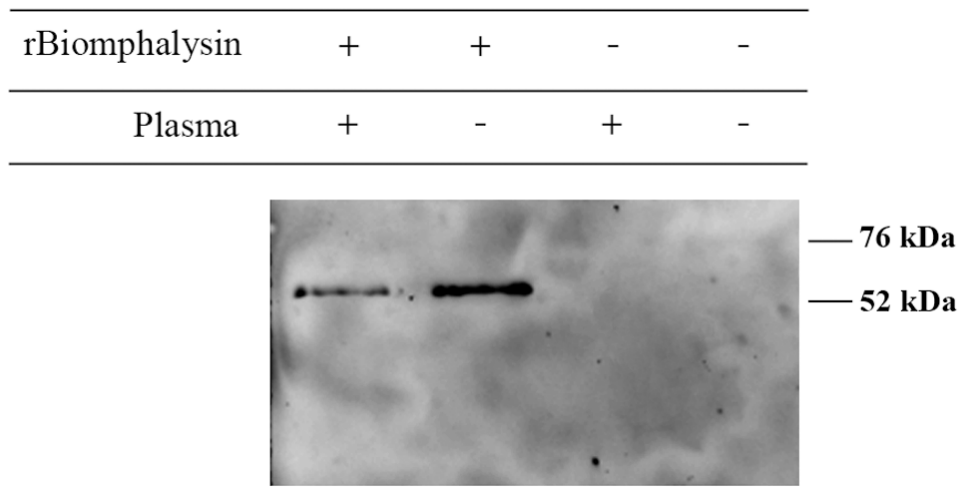
rBiomphalysin binding to the parasite tegument. *In vitro* binding assay was performed on primary sporocysts. rBiomphalysin binding to sporocysts membrane was tested in presence or absence of plasma from snail. After incubation, sporocysts were centrifuged, washed in CBSS, and denatured in Laemmli buffer at 80°C for 10 min. Total lysate of sporocysts were separated by SDS-PAGE and analyzed by Western blot with a monoclonal anti-His_6_ antibody.

Because members of the aerolysin family are known to interact with cell membranes of targeted cells, we assessed binding of rBiomphalysin to sporocysts with or without *B. glabrata* plasma using *in vitro* binding assays and analyzed these assays by Western blotting and immunocytochemistry. Immunocytochemistry clearly showed that rBiomphalysin is able to interact with the parasite tegument, an interaction that is not plasma-dependent (Figures 8 and 9). In addition, we observed a heterogeneous staining pattern composed of dots or aggregates in the membrane (Figure 8) similar to that reported in previous studies on hydralysins [45] and proaerolysin [49].

### Analysis of Biomphalysin mRNA expression and tissue distribution

**Figure 10:**
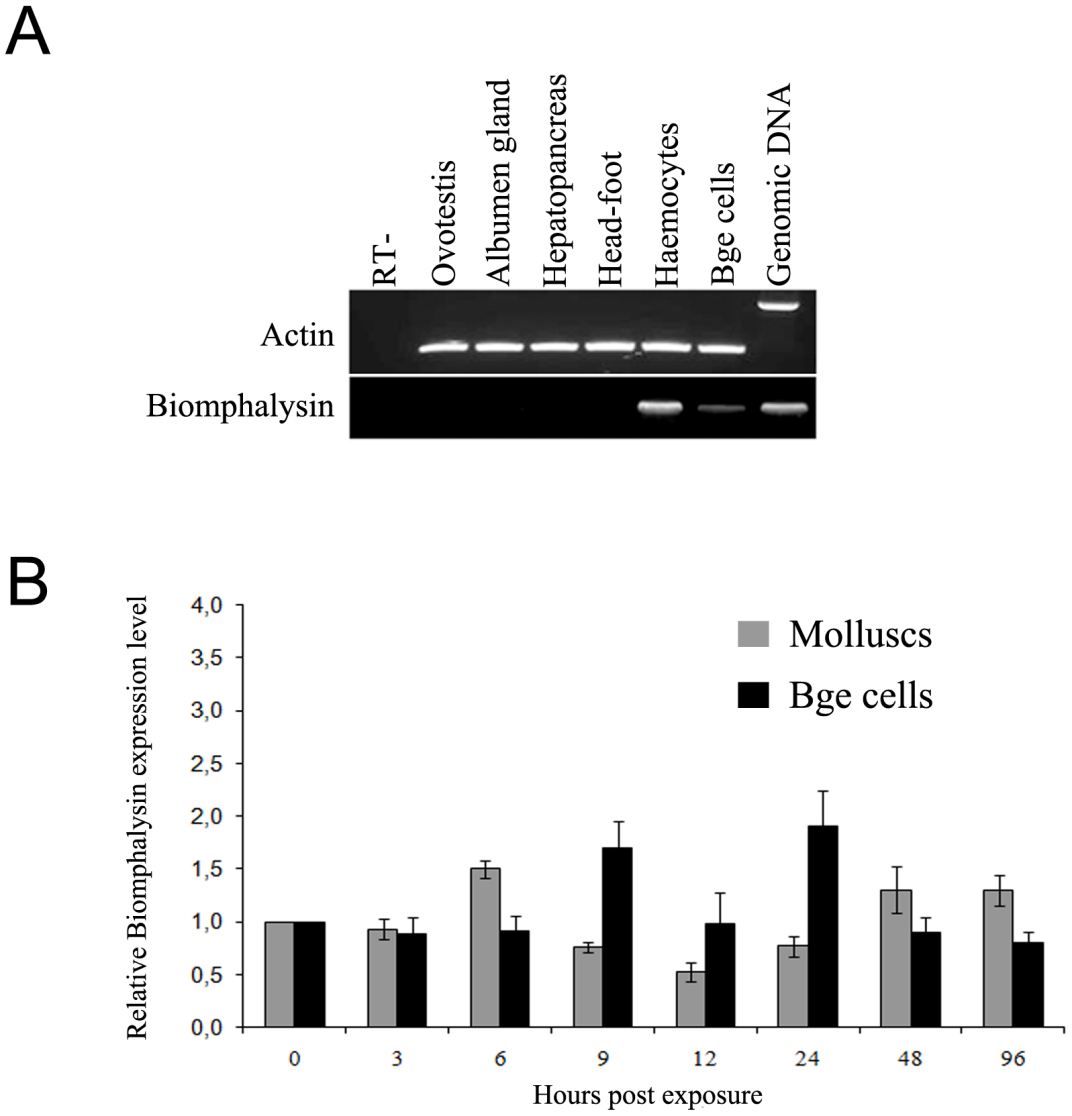
Biomphalysin mRNA tissue distribution and expression in *B. glabrata* in response to *S. mansoni* challenge and in Bge cells in contact with sporocysts. A. Different *B. glabrata* tissues were analyzed by PCR using primers recognizing full-length Biomphalysin; actin was amplified as an endogenous control. Non-reverse transcribed hemocyte RNA was used as a negative control in PCR. B. Biomphalysin transcripts were quantified by Q-PCR in *B. glabrata* challenged with *S. mansoni* for 3, 6, 9, 12, 24, 48 and 96 h and in Bge cells in contact with sporocysts for the same intervals. Biomphalysin mRNA level was normalized to mRNA ribosomal protein S19 transcript abundance using the Roche Applied Science E-method [86]. For graphical representation, the transcription ratio for each challenge was normalized to that obtained for unchallenged snails. Each histogram represents the average value of triplicate experiments ± SD.

The tissue-specific expression of Biomphalysin was investigated by RT-PCR using total RNA extracted from the albumen gland, head-foot, hepato-pancreas, ovotestis organs and hemocytes of *B. glabrata*. β-actin expression was used as a reference. Biomphalysin transcripts were detected only in hemocytes and not in other tissues tested (Figure 10A). Interestingly, Biomphalysin was also detected in cells derived from embryos of *B. glabrata* (Bge cells) maintained in culture. Considering that Bge cells are cultured under aseptic conditions, this latter observation excludes the possibility that Biomphalysin is produced by commensal bacteria present in snail tissues. No size difference was observed between PCR results obtained using genomic DNA or cDNA as a template, indicating that the Biomphalysin gene is intronless. A BLAST search against the Trace Archive Biomphalaria database confirmed the presence of this intronless gene in the snail genome. Considering the suspected anti-schistosomal role of Biomphalysin, we examined whether Biomphalysin expression was modulated by parasite challenge. In these experiments, snails were infected with *S. mansoni* miracidia and Biomphalysin transcripts were quantified by quantitative RT-PCR at different times following exposure (3, 6, 9, 12, 24, 48 and 96 h). Bge cells cultivated in the presence of *in vitro-*transformed sporocysts were also analyzed at the same time points. As shown in Figure 10B, Biomphalysin expression levels did not significantly change after *S. mansoni* challenge compared with uninfected snails or naive Bge cells. These data indicated that Biomphalysin can be considered an immunity-related gene that is constitutively expressed and is not modulated by *S. mansoni* challenge.

## Discussion

In order to identify molecular determinants that play a key role in the interaction between *B. glabrata* and *S. mansoni*, we recently developed an interactome approach that allowed us to discover a factor in *B. glabrata* plasma related to the β-PFT superfamily. This molecule was found in the precipitate containing various molecules from *S. mansoni* (SmPoMucs, glycoprotein k, tetraspanin, chaperone stress proteins, anti-oxidant enzymes) and *B. glabrata* (FREPs, lectin like protein AIF…) [30]. In the present publication, we describe the cloning and functional characterization of this β-PFT of *B. glabrata*, which we have named Biomphalysin.

The full-length cDNA of Biomphalysin was 1972 bp encoding a 572-amino-acid protein with a molecular weight of approximately 63 kDa. Biomphalysin displays low similarities toward database β-PFTs at the primary structure level, but contains an aerolysin domain that is a common core of aerolysin-like β-PFTs [40]. Bioinformatic analysis and protein structure prediction revealed that Biomphalysin contains a large number of β-sheets and has a transmembrane β-barrel domain (Figures 1 and 2). A structural alignment of the aerolysin domain of Biomphalysin with proaerolysin demonstrates structural equivalence, despite the lack of similarity at the amino acid level. Importantly, in addition to these similarities in 3D structure, several key residues involved in β barrel pore formation or receptor binding are conserved. The aerolysins and related toxin family members can be divided into two groups based on their structural shape. A few β-PFTs have two distinct lobes, whereas others, like α toxin, ε toxin and parasporin, have only a single lobe (for a review, see [42]). The common, larger lobe is involved in oligomerization or binding to GPI-anchored receptors, whereas the second, smaller lobe contains a carbohydrate receptor-binding domain. The Biomphalysin smaller lobe, which contains no known domains and displays no identifiable sequence similarities, is an intriguing structure. We postulate that it could be involved in the specificity of Biomphalysin, allowing it to interact with sporocyst antigens or an intermediate molecular partner in *B. glabrata* plasma. Additional studies will be required to address this hypothesis. Interestingly, the phylogenetic tree of aerolysin-like molecules (Figure 4) suggests that Biomphalysin could have been transferred horizontally from bacteria to *B. glabrata*. This hypothesis is strengthened by a recent phylogenetic analysis showing that numerous cross-kingdom horizontal transfers occurred for genes encoding aerolysin-like proteins [71]. We showed that the gene encoding Biomphalysin does not possess intronic regions (Figure 10A), which also argues in favor of a horizontal transfer mechanism. The exclusive expression of Biomphalysin in hemocytes, the immune cells of *B. glabrata*, consolidates the role of Biomphalysin in immunity. Nevertheless, its expression is constitutive and is not modulated by Schistosome infection. Biomphalysin protein was detected in the plasma of uninfected snails and was shown to interact *in vitro* with parasite proteins, suggesting a sentinel role in preventing pathogen invasion [30].

To further characterize Biomphalysin and validate its biological function, we expressed rBiomphalysin using a cell-free protein expression system, a strategy that seems justifiable in view of the lack of predicted glycosylation events during Biomphalysin processing. As described for most toxins related to the aerolysin family, Biomphalysin possessed hemolytic activity. This activity was detectable at 5 nM Biomphalysin and caused complete erythrocyte lysis at a concentration of 90 nM. Surprisingly, co-incubation with *B. glabrata* plasma greatly enhanced the lytic activity of Biomphalysin, enabling 50-fold less recombinant protein to achieve the same activity level. This intriguing result suggests that one or more plasma factors could act on the maturation of Biomphalysin through proteolytic cleavage or by affecting its capacity to bind the target cell membrane. To understand how such cofactor(s) might mediate or enhance Biomphalysin activity, we performed Biomphalysin–sporocyst binding assays in the presence or absence of *B. glabrata* plasma. Analyses of these assays by Western blotting and immunocytochemistry revealed that Biomphalysin binds to the sporocyst membrane in the absence of plasma; moreover, there was no evidence of any cleavage products (Figures 8 and 9). Consistent with this latter observation, an exhaustive analysis of putative cleavage sites failed to detect such a site in the C-terminal region of Biomphalysin. Although the cytolytic effect of most of aerolysin toxins requires proteolytic activation, a similar lack of a proteolytic processing has been previously reported for hydralysin, a cnidarian hemolytic β-PFT [45]. Our results suggest that a plasma factor induces Biomphalysin activity by mediating the conversion of the oligomeric prepore to a functional pore. Thus, this plasma factor could be a chaperone, a type of functional activation that has been described in other models. For example, it has been shown *in vitro* that the prepore-to-pore transition of anthrax toxin is enhanced by an exogenous chaperone, such as GroEL [84]. We speculate that the small lobe of aerolysin could serve as “bait” to recruit the Biomphalysin activity-inducing factor, which remains to be identified. Further investigation is warranted to elucidate the role of the Biomphalysin small lobe.

In conclusion, this report is the first to characterize a mollusk β-PFT that displays antiparasitic activity. This novel β-PFT, which we have named Biomphalysin to reflect its source, shares structural features and activity with aerolysin-like toxins described in numerous bacteria species. The corresponding gene was probably acquired by horizontal transfer, and the cytolytic activity of Biomphalysin is mediated by a plasma factor that remains to be identified.

## References

[1] Doenhoff MJ, Kusel JR, Coles GC, Cioli D (2002) Resistance of Schistosoma mansoni to praziquantel: is there a problem? Trans R Soc Trop Med Hyg 96: 465–469. 12474468 10.1016/s0035-9203(02)90405-0

[2] Melman SD, Steinauer ML, Cunningham C, Kubatko LS, Mwangi IN, et al (2009) Reduced susceptibility to praziquantel among naturally occurring Kenyan isolates of Schistosoma mansoni. PLoS Negl Trop Dis 3: e504. 19688043 10.1371/journal.pntd.0000504 PMC2721635

[3] Adema CM, Hertel LA, Miller RD, Loker ES (1997) A family of fibrinogen-related proteins that precipitates parasite-derived molecules is produced by an invertebrate after infection. Proc Natl Acad Sci U S A 94: 8691–8696. 9238039 10.1073/pnas.94.16.8691 PMC23082

[4] Loker ES, Adema CM, Zhang SM, Kepler TB (2004) Invertebrate immune systems–not homogeneous, not simple, not well understood. Immunol Rev 198: 10–24. 15199951 10.1111/j.0105-2896.2004.0117.x PMC5426807

[5] Hanington PC, Forys MA, Dragoo JW, Zhang SM, Adema CM, et al (2011) Role for a somatically diversified lectin in resistance of an invertebrate to parasite infection. Proc Natl Acad Sci U S A 107: 21087–21092. 10.1073/pnas.1011242107 PMC3000291 21084634

[6] Baeza Garcia A, Pierce RJ, Gourbal B, Werkmeister E, Colinet D, et al (2010) Involvement of the cytokine MIF in the snail host immune response to the parasite Schistosoma mansoni. PLoS Pathog 6 9: e1001115 doi:10.1371/journal.ppat.1001115. 20886098 10.1371/journal.ppat.1001115 PMC2944803

[7] Deleury E, Dubreuil G, Elangovan N, Wajnberg E, Reichhart JM, et al (2012) Specific versus non-specific immune responses in an invertebrate species evidenced by a comparative de novo sequencing study. PLoS One 7: e32512. 22427848 10.1371/journal.pone.0032512 PMC3299671

[8] Guillou F, Mitta G, Galinier R, Coustau C (2007) Identification and expression of gene transcripts generated during an anti-parasitic response in Biomphalaria glabrata. Dev Comp Immunol 31: 657–671. 17166585 10.1016/j.dci.2006.10.001

[9] Ittiprasert W, Miller A, Myers J, Nene V, El-Sayed NM, et al (2010) Identification of immediate response genes dominantly expressed in juvenile resistant and susceptible Biomphalaria glabrata snails upon exposure to Schistosoma mansoni. Mol Biochem Parasitol 169: 27–39. 19815034 10.1016/j.molbiopara.2009.09.009 PMC2785114

[10] Raghavan N, Miller AN, Gardner M, FitzGerald PC, Kerlavage AR, et al (2003) Comparative gene analysis of Biomphalaria glabrata hemocytes pre- and post-exposure to miracidia of Schistosoma mansoni. Mol Biochem Parasitol 126: 181–191. 12615317 10.1016/s0166-6851(02)00272-4

[11] Lockyer AE, Spinks JN, Walker AJ, Kane RA, Noble LR, et al (2007) Biomphalaria glabrata transcriptome: identification of cell-signalling, transcriptional control and immune-related genes from open reading frame expressed sequence tags (ORESTES). Dev Comp Immunol 31: 763–782. 17208299 10.1016/j.dci.2006.11.004 PMC1871615

[12] Nowak TS, Woodards AC, Jung Y, Adema CM, Loker ES (2004) Identification of transcripts generated during the response of resistant Biomphalaria glabrata to Schistosoma mansoni infection using suppression subtractive hybridization. J Parasitol 90: 1034–1040. 15562603 10.1645/GE-193R1

[13] Bouchut A, Coustau C, Gourbal B, Mitta G (2007) Compatibility in the Biomphalaria glabrata/Echinostoma caproni model: new candidate genes evidenced by a suppressive subtractive hybridization approach. Parasitology 134: 575–588. 17096871 10.1017/S0031182006001673

[14] Bouchut A, Roger E, Coustau C, Gourbal B, Mitta G (2006) Compatibility in the Biomphalaria glabrata/Echinostoma caproni model: potential involvement of adhesion genes. Int J Parasitol 36: 175–184. 16310790 10.1016/j.ijpara.2005.09.009

[15] Bouchut A, Sautiere PE, Coustau C, Mitta G (2006) Compatibility in the *Biomphalaria glabrata/Echinostoma caproni* model: Potential involvement of proteins from hemocytes revealed by a proteomic approach. Acta Tropica 98: 234–246. 16792992 10.1016/j.actatropica.2006.05.007

[16] Lockyer AE, Spinks J, Kane RA, Hoffmann KF, Fitzpatrick JM, et al (2008) Biomphalaria glabrata transcriptome: cDNA microarray profiling identifies resistant- and susceptible-specific gene expression in haemocytes from snail strains exposed to Schistosoma mansoni. BMC Genomics 9: 634. 19114004 10.1186/1471-2164-9-634 PMC2631019

[17] Vergote D, Bouchut A, Sautiere PE, Roger E, Galinier R, et al (2005) Characterisation of proteins differentially present in the plasma of *Biomphalaria glabrata* susceptible or resistant to *Echinostoma caproni* . Int J Parasitol 35: 215–224. 15710442 10.1016/j.ijpara.2004.11.006

[18] Roger E, Gourbal B, Grunau C, Pierce RJ, Galinier R, et al (2008) Expression analysis of highly polymorphic mucin proteins (Sm PoMuc) from the parasite Schistosoma mansoni. Mol Biochem Parasitol 157: 217–227. 18187213 10.1016/j.molbiopara.2007.11.015

[19] Roger E, Grunau C, Pierce RJ, Hirai H, Gourbal B, et al (2008) Controlled Chaos of Polymorphic Mucins in a Metazoan Parasite (Schistosoma mansoni) Interacting with Its Invertebrate Host (Biomphalaria glabrata). PLoS Negl Trop Dis 2: e330. 19002242 10.1371/journal.pntd.0000330 PMC2576457

[20] Roger E, Mitta G, Mone Y, Bouchut A, Rognon A, et al (2008) Molecular determinants of compatibility polymorphism in the Biomphalaria glabrata/Schistosoma mansoni model: New candidates identified by a global comparative proteomics approach. Mol Biochem Parasitol 157: 205–216. 18083248 10.1016/j.molbiopara.2007.11.003

[21] Mone Y, Gourbal B, Duval D, Du Pasquier L, Kieffer-Jaquinod S, et al (2010) A large repertoire of parasite epitopes matched by a large repertoire of host immune receptors in an invertebrate host/parasite model. PLoS Negl Trop Dis 4. 10.1371/journal.pntd.0000813 PMC2935394 20838648

[22] Mitta G, Adema CM, Gourbal B, Loker ES, Theron A (2012) Compatibility polymorphism in snail/schistosome interactions: From field to theory to molecular mechanisms. Developmental & Comparative Immunology 37: 1–8. 21945832 10.1016/j.dci.2011.09.002 PMC3645982

[23] Hahn UK, Bender RC, Bayne CJ (2000) Production of reactive oxygen species by hemocytes of Biomphalaria glabrata: carbohydrate-specific stimulation. Dev Comp Immunol 24: 531–541. 10831788 10.1016/s0145-305x(00)00017-3

[24] Hahn UK, Bender RC, Bayne CJ (2001) Killing of Schistosoma mansoni sporocysts by hemocytes from resistant Biomphalaria glabrata: role of reactive oxygen species. J Parasitol 87: 292–299. 11318558 10.1645/0022-3395(2001)087[0292:KOSMSB]2.0.CO;2

[25] Bender RC, Broderick EJ, Goodall CP, Bayne CJ (2005) Respiratory burst of *Biomphalaria glabrata* hemocytes: *Schistosoma mansoni*-resistant snails produce more extracellular H2O2 than susceptible snails. J Parasitol 91: 275–279. 15986600 10.1645/GE-415R

[26] Bender RC, Goodall CP, Blouin MS, Bayne CJ (2007) Variation in expression of Biomphalaria glabrata SOD1: a potential controlling factor in susceptibility/resistance to Schistosoma mansoni. Dev Comp Immunol 31: 874–878. 17292470 10.1016/j.dci.2006.12.005

[27] Goodall CP, Bender RC, Broderick EJ, Bayne CJ (2004) Constitutive differences in Cu/Zn superoxide dismutase mRNA levels and activity in hemocytes of *Biomphalaria glabrata* (Mollusca) that are either susceptible or resistant to *Schistosoma mansoni* (Trematoda). Molecular and Biochemical Parasitology 137: 321–328. 15383302 10.1016/j.molbiopara.2004.06.011

[28] Mone Y, Ribou AC, Cosseau C, Duval D, Theron A, et al (2011) An example of molecular co-evolution: reactive oxygen species (ROS) and ROS scavenger levels in Schistosoma mansoni/Biomphalaria glabrata interactions. Int J Parasitol 41: 721–730. 21329695 10.1016/j.ijpara.2011.01.007

[29] Mitta G, Galinier R, Tisseyre P, Allienne JF, Girerd-Chambaz Y, et al (2005) Gene discovery and expression analysis of immune-relevant genes from Biomphalaria glabrata hemocytes. Developmental & Comparative Immunology 29: 393–407. 15707661 10.1016/j.dci.2004.10.002

[30] Moné Y, Gourbal B, Duval D, Du Pasquier L, Kieffer-Jaquinod S, et al (2010) A Large Repertoire of Parasite Epitopes Matched by a Large Repertoire of Host Immune Receptors in an Invertebrate Host/Parasite Model. PLoS Negl Trop Dis 4: e813. 20838648 10.1371/journal.pntd.0000813 PMC2935394

[31] Bernheimer AW, Avigad LS (1974) Partial Characterization of Aerolysin, a Lytic Exotoxin from Aeromonas hydrophila. pp. 1016–1021. 10.1128/iai.9.6.1016-1021.1974 PMC414926 4208526

[32] Husslein V, Huhle B, Jarchau T, Lurz R, Goebel W, et al (1988) Nucleotide sequence and transcriptional analysis of the aerCaerA region of Aeromonas sobria encoding aerolysin and its regulatory region. Molecular Microbiology 2: 507–517. 2459581 10.1111/j.1365-2958.1988.tb00057.x

[33] Ballard J, Crabtree J, Roe BA, Tweten RK (1995) The primary structure of Clostridium septicum alpha-toxin exhibits similarity with that of Aeromonas hydrophila aerolysin. Infect Immun 63: 340–344. 7806374 10.1128/iai.63.1.340-344.1995 PMC172997

[34] Hunter SE, Clarke IN, Kelly DC, Titball RW (1992) Cloning and nucleotide sequencing of the Clostridium perfringens epsilon-toxin gene and its expression in Escherichia coli. pp. 102–110. 10.1128/iai.60.1.102-110.1992 PMC257509 1729175

[35] Priest FG, Ebdrup L, Zahner V, Carter PE (1997) Distribution and characterization of mosquitocidal toxin genes in some strains of Bacillus sphaericus. Appl Environ Microbiol 63: 1195–1198. 9097416 10.1128/aem.63.4.1195-1198.1997 PMC168413

[36] Akiba T, Abe Y, Kitada S, Kusaka Y, Ito A, et al (2009) Crystal Structure of the Parasporin-2 Bacillus thuringiensis Toxin That Recognizes Cancer Cells. Journal of Molecular Biology 386: 121–133. 19094993 10.1016/j.jmb.2008.12.002

[37] Okumura S, Saitoh H, Ishikawa T, Mizuki E, Inouye K, et al.. (2008) Identification and characterization of a novel cytotoxic protein, parasporin-4, produced by Bacillus thuringiensis A1470 strain. Biotechnology Annual Review: Elsevier. pp. 225–252. 10.1016/S1387-2656(08)00009-4 18606366

[38] Opota O, Vallet-Gély I, Vincentelli R, Kellenberger C, Iacovache I, et al (2011) Monalysin, a novel ß-pore-forming toxin from the Drosophila pathogen Pseudomonas entomophila, contributes to host intestinal damage and lethality. PLoS Pathog 7: e1002259. 21980286 10.1371/journal.ppat.1002259 PMC3182943

[39] Macpherson H, Bergh Ø, Birkbeck TH (2012) An aerolysin-like enterotoxin from Vibrio splendidus may be involved in intestinal tract damage and mortalities in turbot, Scophthalmus maximus (L.), and cod, Gadus morhua L., larvae. J Fish Dis 35: 153–167. 22233514 10.1111/j.1365-2761.2011.01331.x

[40] Szczesny P, Iacovache I, Muszewska A, Ginalski K, van der Goot FG, et al (2011) Extending the Aerolysin Family: From Bacteria to Vertebrates. PLoS ONE 6: e20349. 21687664 10.1371/journal.pone.0020349 PMC3110756

[41] Rossjohn J, Feil SC, McKinstry WJ, Tsernoglou D, Van Der Goot G, et al (1998) Aerolysine : A Paradigm for Membrane Insertion of Beta-Sheet Protein Toxins? Journal of Structural Biology 121: 92–100. 9615432 10.1006/jsbi.1997.3947

[42] Knapp O, Stiles B, Popoff MR (2010) The Aerolysin Like Toxin Family of cytolitic, Pore Forming Toxins. The open toxinology Journal 3: 53–68.

[43] Jonas D, Schultheis B, Klas C, Krammer PH, Bhakdi S (1993) Cytocidal effects of Escherichia coli hemolysin on human T lymphocytes. pp. 1715–1721. 10.1128/iai.61.5.1715-1721.1993 PMC280756 8478059

[44] Nelson K, Brodsky R, Buckley J (1999) Channels formed by subnanomolar concentrations of the toxin aerolysin trigger apoptosis of T lymphomas. Cell Microbiol 1: 69–74. 11207542 10.1046/j.1462-5822.1999.00009.x

[45] Sher DJ, Fishman Y, Zhang M, Lebendiker M, Gaathon A, et al (2005) Hydralysins: a new category of beta-pore-forming toxins in cnidaria. Characterization and preliminary structure-function analysis. J Biol Chem 280: 22847–22855. 15824108 10.1074/jbc.M503242200

[46] Castro-Faria-Neto HC, Martins MA, Bozza PT, Perez SAC, Correa-Da-Silva ACV, et al (1991) Pro-inflammatory activity of enterolobin: A haemolytic protein purified from seeds of the Brazilian tree Enterolobium contortisiliquum. Toxicon 29: 1143–1150. 1796477 10.1016/0041-0101(91)90211-9

[47] Sousa MV, Richardson M, Fontes W, Morhy L (1994) Homology between the seed cytolysin enterolobin and bacterial aerolysins. J Protein Chem 13: 659–667. 7710657 10.1007/BF01886950

[48] Iacovache I, van der Goot FG, Pernot L (2008) Pore formation: An ancient yet complex form of attack. Biochimica et Biophysica Acta (BBA) - Biomembranes 1778: 1611–1623. 18298943 10.1016/j.bbamem.2008.01.026

[49] Abrami L, Fivaz M, Glauser PE, Parton RG, van der Goot FG (1998) A pore-forming toxin interact with a GPI-anchored protein and causes vacuolation of the endoplasmic reticulum. J Cell Biol 140: 525–540. 9456314 10.1083/jcb.140.3.525 PMC2140172

[50] Rossjohn J, Buckley JT, Hazes B, Murzin AG, Read RJ, et al (1997) Aerolysin and pertussis toxin share a common receptor-binding domain. EMBO J 16: 3426–3434. 9218785 10.1093/emboj/16.12.3426 PMC1169968

[51] Diep DB, Nelson KL, Lawrence TS, Sellman BR, Tweten RK, et al (1999) Expression and properties of an aerolysin–Clostridium septicum alpha toxin hybrid protein. Molecular Microbiology 31: 785–794. 10048023 10.1046/j.1365-2958.1999.01217.x

[52] Melton-Witt JA, Bentsen LM, Tweten RK (2006) Identification of Functional Domains of Clostridium septicum Alpha Toxinâ€. Biochemistry 45: 14347–14354. 17128973 10.1021/bi061334p PMC2561313

[53] Wilmsen H, Leonard K, Ticheaar W, Buckley J, Pattus F (1992) The aerolysin membrane channel is formed by heptamerization of the monomer. EMBO J 11: 2457–2463. 1378390 10.1002/j.1460-2075.1992.tb05310.x PMC556720

[54] MacKenzie CR, Hirama T, Buckley JT (1999) Analysis of receptor binding by the channel-forming toxin aerolysin using surface plasmon resonance. J Biol Chem 274: 22604–22609. 10428840 10.1074/jbc.274.32.22604

[55] Iacovache I, Paumard P, Scheib H, Lesieur C, Sakai N, et al (2006) A rivet model for channel formation by aerolysin-like pore-forming toxins. EMBO J 25: 457–466. 16424900 10.1038/sj.emboj.7600959 PMC1383540

[56] Howard SP, Buckley JT (1985) Activation of the hole-forming toxin aerolysin by extracellular processing. pp. 336–340. 10.1128/jb.163.1.336-340.1985 PMC219118 3891735

[57] Garland WJ, Buckley JT (1988) The cytolytic toxin aerolysin must aggregate to disrupt erythrocytes, and aggregation is stimulated by human glycophorin. pp. 1249–1253. 10.1128/iai.56.5.1249-1253.1988 PMC259800 3281905

[58] Song T, Toma C, Nakasone N, Iwanaga M (2004) Aerolysin is activated by metalloprotease in Aeromonas veronii biovar sobria. J Med Microbiol 53 6: 477–482. 15150325 10.1099/jmm.0.05405-0

[59] Bech N, Beltran S, Portela J, Rognon A, Allienne J-Fo, et al Follow-up of the genetic diversity and snail infectivity of a Schistosoma mansoni strain from field to laboratory. Infection, Genetics and Evolution 10: 1039–1045. 10.1016/j.meegid.2010.06.012 20601175

[60] Theron A, Pages JR, Rognon A (1997) Schistosoma mansoni: distribution patterns of miracidia among Biomphalaria glabrata snail as related to host susceptibility and sporocyst regulatory processes. Exp Parasitol 85: 1–9. 9024196 10.1006/expr.1996.4106

[61] Yoshino T, Laursen J (1995) Production of Schistosoma mansoni daughter sporocysts from mother sporocysts maintained in synxenic culture with Biomphalaria glabrata embryonic (Bge) cells. J Parasitol 81: 714–722. 7472861

[62] Hansen E (1976) Application of tissue culture of a pulmonate snail to culture of larval of Schistosoma mansoni. New York: Academic Press: 87–97.

[63] Sambrook J, Fritsch EF, Maniatis T (1989) Molecular Cloning: a laboratory manual. 2 ed. New York: Cold Spring Harbor Laboratory Press.

[64] Galinier R, Roger E, Sautiere P, Aumelas A, Banaigs B, et al (2009) Halocyntin and papillosin, two new antimicrobial peptides isolated from hemocytes of the solitary tunicate, Halocynthia papillosa. Journal of Peptide Science 15: 48–55. 19085906 10.1002/psc.1101

[65] Mattos AC, Kusel JR, Pimenta PF, Coelho PM (2006) Activity of praziquantel on in vitro transformed Schistosoma mansoni sporocysts. Mem Inst Oswaldo Cruz 101 Suppl 1: 283–287. 17308783 10.1590/s0074-02762006000900044

[66] Bagos PG, Liakopoulos TD, Spyropoulos IC, Hamodrakas SJ (2004) PRED-TMBB: a web server for predicting the topology of Î^2^-barrel outer membrane proteins. pp. W400–W404. 10.1093/nar/gkh417 PMC441555 15215419

[67] Bagos P, Liakopoulos T, Spyropoulos I, Hamodrakas S (2004) A Hidden Markov Model method, capable of predicting and discriminating beta-barrel outer membrane proteins. BMC Bioinformatics (5): 29. 15070403 10.1186/1471-2105-5-29 PMC385222

[68] Zhang Y, Skolnick J (2005) TM-align: a protein structure alignment algorithm based on the TM-score. pp. 2302–2309. 10.1093/nar/gki524 PMC1084323 15849316

[69] Zhang Y (2008) I-TASSER server for protein 3D structure prediction. BMC Bioinformatics (9): 40. 18215316 10.1186/1471-2105-9-40 PMC2245901

[70] Roy A, Kucukural A, Zhang Y (2010) I-TASSER: a unified platform for automated protein structure and function prediction. Nature Protocols (5): 725–738. 20360767 10.1038/nprot.2010.5 PMC2849174

[71] Moran Y, Fredman D, Szczesny P, Grynberg M, Technau U (2012) Recurrent Horizontal Transfer of Bacterial Toxin Genes to Eukaryotes. Mol Biol Evol 10.1093/molbev/mss089 PMC3424411 22411854

[72] Capella-Gutierrez S, Silla-Martinez J, TG T (2009) trimAl: a tool for automated alignment trimming in large-scale phylogenetic analyses. Bioinformatics 25: 1972–1973. 19505945 10.1093/bioinformatics/btp348 PMC2712344

[73] Abascal F, Zardoya R, Posada D (2005) ProtTest: Selection of best-fit models of protein evolution. Bioinformatics 21: 2104–2105. 15647292 10.1093/bioinformatics/bti263

[74] Ronquist F, Huelsenbeck JP (2003) MrBayes 3: Bayesian phylogenetic inference under mixed models. Bioinformatics 19: 1572–1574. 12912839 10.1093/bioinformatics/btg180

[75] Guindon S, Gascuel O (2003) A simple, fast, and accurate algorithm to estimate large phylogenies by maximum likelihood. Systematic Biology 52: 696–704. 14530136 10.1080/10635150390235520

[76] Fivaz M, Abrami L, Tsitrin Y, Goot FVd (2001) Aerolysin from Aeromonas hydrophila and related toxins. Curr Top Microbiol Immunol 257: 35–52. 11417121 10.1007/978-3-642-56508-3_3

[77] Cole AR, Gibert M, Popoff M, Moss DS, Titball RW, et al (2004) Clostridium perfringens epsilon-toxin shows structural similarity to the pore-forming toxin aerolysin. Nat Struct Mol Biol 11: 797–798. 15258571 10.1038/nsmb804

[78] Tsitrin Y, Morton CJ, El Bez C, Paumard P, Velluz M-C, et al (2002) Conversion of a transmembrane to a water-soluble protein complex by a single point mutation. Nat Struct Mol Biol 9: 729–733. 10.1038/nsb839 12219082

[79] Green M, Buckley J (1990) Site-directed mutagenesis of the hole-forming toxin aerolysin: studies on the roles of histidines in receptor binding and oligomerization of the monomer. Biochemistry 29: 2177–2180. 2158347 10.1021/bi00460a031

[80] Wilmsen HU, Buckley JT, Pattus F (1991) Site-directed mutagenesis at histidines of aerolysin from Aeromonas hydrophila: a lipid planar bilayer study. Mol Microbiol 5: 2745–2751. 1723472 10.1111/j.1365-2958.1991.tb01983.x

[81] Buckley JT, Wilmsen HU, Lesieur C, Schultze A, Pattus F, et al (1995) Protonation of His-132 promotes oligomerization of the channel-forming toxin Aerolysin. Biochemistry 34: 16450–16455. 8845373 10.1021/bi00050a028

[82] van der Goot FGPF, Wong KR, Buckley JT (1993) Oligomerization of the channel-forming toxin aerolysin precedes insertion into lipid bilayers. Biochemistry 32: 2636–2642. 7680572 10.1021/bi00061a023

[83] Abrami L, Fivaz M, Decroly E, Seidah NG, Jean Fo, et al (1998) The Pore-forming Toxin Proaerolysin Is Activated by Furin. 273: 32656–32661. 10.1074/jbc.273.49.32656 9830006

[84] Katayama H, Janowiak BE, Brzozowski M, Juryck J, Falke S, et al (2008) GroEL as a molecular scaffold for structural analysis of the anthrax toxin pore. Nat Struct Mol Biol 15: 754–760. 18568038 10.1038/nsmb.1442 PMC2504863

[85] Kyte J, Doolittle RF (1982) A simple method for displaying the hydropathic character of a protein. Journal of Molecular Biology 157: 105–132. 7108955 10.1016/0022-2836(82)90515-0

[86] Tellmann G, Geulen O (2006) LightCycler 480 Real-Time PCR system: Innovative solutions for relative quantification. Biochemica 16–18.

